# Dynamic machine-learning model for early prediction of intraoperative hypotension using 5-minute perioperative time-series data: a multicenter retrospective study

**DOI:** 10.3389/fmed.2026.1884174

**Published:** 2026-09-30

**Authors:** Bo Long, Yifeng Ye, Hang Xing, Lijun Yang

**Affiliations:** 1 Department of Anesthesiology, The Second Affiliated Hospital of Fujian University of Traditional Chinese Medicine, Fuzhou, Fujian, China; 2 Department of Anesthesiology, Fujian Maternity and Child Health Hospital College of Clinical Medicine for Obstetrics & Gynecology and Pediatrics, Fujian Medical University, Fuzhou, Fujian, China; 3 Department of Anesthesiology, The First Affiliated Hospital of Xiamen University, School of Medicine, Xiamen University, Xiamen, China; 4 Department of Pediatrics, Women & Infants Hospital of Rhode Island, Warren Alpert Medical School of Brown University, Providence, RI, United States

**Keywords:** dynamic prediction, intraoperative hypotension, machine learning, non-cardiac surgery, time-series analysis

## Abstract

**Background:**

Intraoperative hypotension (IOH) is a common and potentially preventable complication strongly associated with postoperative acute kidney injury, myocardial injury, and mortality. Current approaches to IOH management remain largely reactive, and existing prediction tools have variable clinical utility. This study aimed to develop and externally validate a dynamic machine-learning model using routinely collected perioperative data to perform one-step-ahead prediction of intraoperative hypotension on a regularized 5-minute intraoperative grid.

**Methods:**

This retrospective multicenter study included 702 adults undergoing non-cardiac surgery under general anesthesia for model development and internal validation and an independent external cohort of 248 patients. A dynamic prediction framework was constructed using routinely collected preoperative variables and intraoperative 5-minute time-series data. Individualized baseline features and temporal predictors were derived from rolling observation windows. Four machine-learning algorithms were compared using a fully nested grouped cross-validation framework with external validation. Model performance was assessed by discrimination, calibration, decision-curve analysis, and SHapley Additive exPlanations (SHAP)-based interpretability.

**Results:**

All four machine-learning models demonstrated good discrimination in the primary fully nested case-grouped internal validation, with AUROC values ranging from 0.842 to 0.857. Random forest achieved the highest AUROC (0.857, 95% CI 0.841–0.868), the highest AUPRC (0.413, 95% CI 0.367–0.452), and extra trees achieved the lowest Brier score (0.093, 95% CI 0.088–0.098). In the independent external validation cohort, random forest remained the best-performing model, yielding an AUROC of 0.894 (95% CI 0.875–0.907), an AUPRC of 0.540 (95% CI 0.457–0.596), and a Brier score of 0.092 (95% CI 0.083–0.102). SHAP analysis consistently identified MAP-derived features, including current MAP, recent hypotensive burden, recent MAP measurements, relative MAP reduction, and MAP variability, as the most influential predictors across all four models.

**Conclusions:**

A dynamic machine-learning model using routinely collected perioperative time-series data accurately predicted MAP <65 mmHg at the next scheduled 5-minute intraoperative time point in an independent external cohort. These findings support further evaluation of this short-horizon risk stratification model in prospective clinical workflow studies.

## Introduction

Intraoperative hypotension (IOH) represents one of the most pervasive and clinically consequential challenges in modern perioperative care. Defined most commonly as a mean arterial pressure (MAP) below 65 mmHg, IOH is encountered with alarming frequency during non-cardiac surgery (1). A large retrospective analysis of 127,095 non-cardiac surgical cases in community anesthesia practice reported that 29% of patients experienced MAP <65 mmHg for at least 15 cumulative minutes, with an overall mean duration of 12.4 min below this threshold (2). Even among patients and settings traditionally considered “low risk,” IOH remains a pervasive phenomenon, with substantial inter-clinician variability in incidence even after risk adjustment, underscoring its nature as a modifiable quality improvement target (2).

The clinical significance of IOH extends far beyond transient hemodynamic perturbation. A landmark meta-analysis by Walsh and colleagues demonstrated that intraoperative MAP values below 60 mmHg for as little as 1–5 min were independently associated with an increased risk of postoperative acute kidney injury (AKI) [odds ratio (OR), 1.13; 95% CI, 1.04–1.23] and myocardial injury (OR, 1.16; 95% CI, 1.01–1.33), with risk escalating in a duration-dependent manner (3). In a separate meta-analysis encompassing 15 observational studies, An et al. corroborated these findings, demonstrating that MAP <60 mmHg for >10 min was associated with an OR of 1.35 (95% CI, 1.10–1.67) for AKI and 1.43 (95% CI, 1.18–1.72) for myocardial injury (4). The Perioperative Quality Initiative consensus statement has affirmed that intraoperative MAP values below 60–70 mmHg are associated with myocardial injury, AKI, and death, with injury severity being a function of both hypotension severity and cumulative duration (5). More recently, data from off-pump coronary artery bypass grafting surgery have confirmed MAP >65 mmHg as a significant independent protective factor for AKI (OR, 0.408; *P* = 0.008) (6). These converging lines of evidence establish IOH not merely as a hemodynamic epiphenomenon but as a direct, duration-dependent contributor to end-organ injury.

Despite the well-characterized consequences of IOH, current clinical management remains predominantly reactive rather than anticipatory. Traditional blood pressure monitoring, whether via intermittent noninvasive cuff measurements or continuous invasive arterial lines, alerts clinicians only after hypotension has already occurred (7). The therapeutic window between hemodynamic detection and clinical intervention is often compressed, resulting in preventable cumulative hypotensive exposure.

In response to this unmet need, machine learning (ML) approaches to IOH prediction have emerged as a paradigm-shifting innovation in perioperative medicine. The commercially available Hypotension Prediction Index (HPI), developed by Hatib et al. using a logistic regression model trained on high-fidelity arterial waveform features, demonstrated an AUROC of 0.95 (sensitivity 88%, specificity 87%) for predicting hypotension 15 min in advance (8, 9). Clinical implementation of HPI-guided hemodynamic management protocols has been associated with significant reductions in the time-weighted average of hypotension during non-cardiac surgery (10). However, the proprietary nature of HPI, its reliance on invasive arterial waveform data which limits applicability to the subset of surgical patients with arterial catheterization and recent concerns regarding its potential overcorrelation with concurrent MAP have prompted calls for independent, transparent model development (11). A systematic review and meta-analysis of 22 studies by Shirmohamadi et al. found that while HPI demonstrated pooled sensitivity and specificity of 83% each (AUC 0.90), its clinical advantage over standard MAP-based monitoring remains uncertain (12). A scoping review by Koh et al. (26 studies, *N* = 48,707 patients) confirmed that 92.3% of ML studies for IOH prediction focused on non-cardiac surgery, with HPI achieving a median AUROC of 0.912 (range, 0.896–0.930) (13).

Parallel to commercial HPI development, academic efforts have explored diverse modeling strategies. Lee et al. proposed a systematic feature engineering framework using random forest on invasive blood pressure waveforms, achieving accuracy of 0.974 and precision of 0.904 for 5-minute-ahead predictions (14). Hwang et al. developed an interpretable deep learning model forecasting hypotension 10 min in advance using 90-second arterial blood pressure segments, achieving internal and external validation AUROCs of 0.9145 and 0.9035, respectively (15). Zhu et al. developed a Transformer-based deep learning model trained on 319,699 surgical cases, achieving 5-minute prediction AUROC of 0.904 with recall ≥88.3% and external validation across international datasets (16). The Hybrid Multi-Factor network proposed by Cheng et al. formulated IOH prediction as a dynamic sequence forecasting task, capturing both temporal dependencies and physiological non-stationarity (17). Most recently, Garmendia et al. (2023) demonstrated that personalized prediction using LSTM models on patient clusters defined by baseline MAP trends improved prediction AUC from 0.83 to 0.87 (18). These advances collectively underscore the feasibility and promise of ML-driven IOH prediction.

Notwithstanding these important advances, several critical gaps persist in the literature. First, many existing models rely exclusively on high-fidelity arterial waveform data, limiting their generalizability to patients monitored with standard noninvasive equipment who constitute the majority of surgical cases. Second, most published models are static or utilize relatively simplistic temporal representations, failing to exploit the rich trajectory information embedded in continuous intraoperative monitoring data. Third, while the importance of individualized blood pressure management is increasingly recognized (19, 20), most prediction models rely solely on population-level thresholds without incorporating patient-specific physiological baselines. Fourth, comparative evaluations of discrimination, calibration, and clinical utility using transparent, interpretable modeling approaches remain underreported outside of proprietary commercial platforms.

The present study was designed to address these limitations through the development and validation of a dynamic machine-learning model that incorporates five key methodological innovations: (1) a structured time-series framework with 5-minute resolution organized into rolling 10-minute observation windows; (2) individualized intraoperative baseline estimation capturing patient-specific deviation from early stable physiology; (3) comprehensive feature engineering encompassing current-value, lagged, baseline-relative, trend, variability, and hypotensive burden features; (4) rigorous feature selection via LASSO logistic regression followed by comparison of multiple supervised classification algorithms; and (5) thorough evaluation of discrimination, calibration, and interpretability using SHapley Additive exPlanations (SHAP) analysis. The model was externally validated in an independent cohort from a separate tertiary-care hospital to assess generalizability.

## Methods

### Study design and population

This retrospective observational study included adult patients (age ≥18 years) undergoing non-cardiac surgery under general anesthesia. Data were collected from two independent tertiary-care hospitals to enhance generalizability. The cohort from The Second Affiliated Hospital of Fujian University of Traditional Chinese Medicine (SHFU) was used for model development and internal validation, while the cohort from The First Affiliated Hospital of Xiamen University (FHXU) (*n* = 248) served as an independent external validation dataset. The study protocol was approved by the Institutional Review Board of SHFU, with a waiver of informed consent due to the retrospective nature of the study. All procedures were conducted in accordance with the principles of the Declaration of Helsinki.

Patients were eligible if they met all of the following criteria: age ≥18 years; receipt of general anesthesia; first surgery during the hospitalization; and undergoing non-cardiac surgery without complex combined procedures. Patients were excluded if they had a surgical duration <60 min; had substantial missing data or unresolvable misalignment in intraoperative time-series records; lacked a usable baseline window (first 20 min post-induction); or exhibited persistent hypotension during the first 20 min after induction, defined as four consecutive 5-minute MAP values <65 mmHg. The flowchart of study population selection was shown in Figure 1 (SHFU) and Supplementary Figure S1 (FHXU). Supplementary Material S1 outlines the full analytic pipeline.

**Figure 1:**
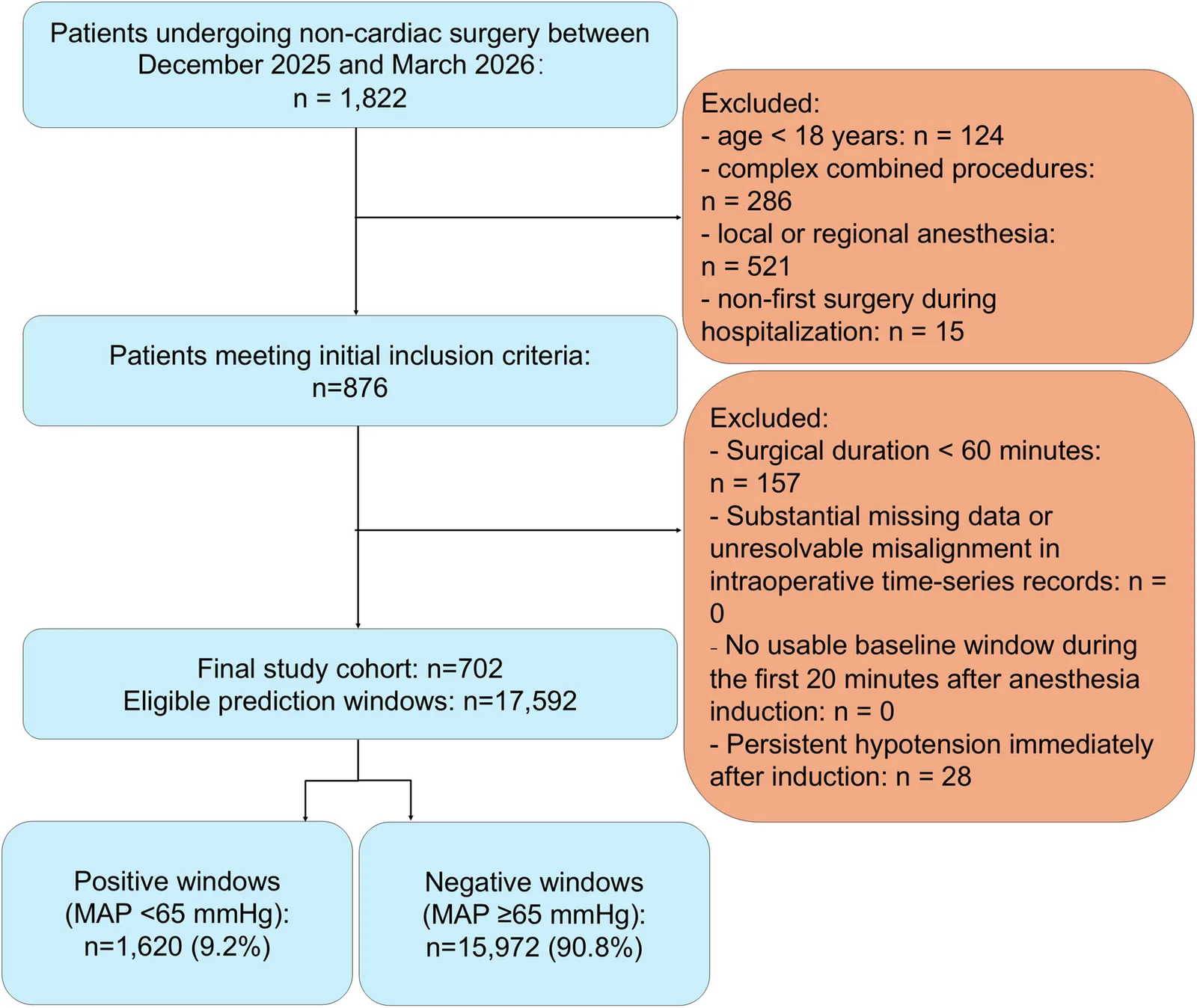
Study population selection flowchart. Flowchart showing inclusion and exclusion criteria applied to the SHFU cohort, resulting in the final analytic samples for model development and internal validation.

### Data sources and Variable collection

The perioperative records were extracted from the hospital electronic medical record system. Two structured datasets were used for model development: a baseline dataset containing preoperative patient-level variables and an intraoperative dataset containing repeated physiologic measurements. The baseline dataset included demographic characteristics (age, sex, body mass index), preoperative vital signs (systolic blood pressure, diastolic blood pressure, MAP, heart rate), social history (smoking, alcohol), comorbidities (hypertension, coronary artery disease, heart failure, arrhythmia, myocardial infarction, stroke, diabetes mellitus, chronic kidney disease, chronic respiratory disease, malignancy), laboratory measurements (hemoglobin, hematocrit, white blood cell count, platelet count, creatinine, blood urea nitrogen, sodium, potassium, chloride, calcium, albumin, glucose, prothrombin time, INR, APTT), surgical context (emergency status, surgical type), and preoperative medication exposure (beta-blockers, ACE inhibitors/angiotensin receptor blockers, diuretics). The intraoperative dataset comprised serial measurements of MAP, HR, SpO₂, EtCO₂, vasopressor exposure, elapsed operative time, and elapsed anesthesia time. MAP, HR, SpO₂, EtCO₂ measurements were obtained from routine non-invasive oscillometric blood pressure monitoring performed in both cohorts (BeneVision N1, Mindray, China). All observations were aligned to a regular 5-minute time grid. Predictor variables were constructed from the current and two preceding observations (t, t−5, and t−10), with the earliest prediction generated 10 min after anesthesia induction. Because multiple prediction windows were generated from each surgical case, all analyses were performed using case-grouped resampling to prevent information leakage.

### Individualized intraoperative baseline

To capture patient-specific physiologic reference levels, individualized intraoperative baselines were estimated during the early anesthetic period (0–20 min after anesthesia initiation). This interval was selected as a pragmatic, standardized early intraoperative reference window rather than a perfectly invariant physiologic steady state, and was considered usable when sufficient early observations were available to derive baseline features on the regularized 5-minute grid. For each variable (MAP, HR, SpO₂, EtCO₂), the mean of available observations within this interval was defined as the individualized baseline. Standard deviations within the same interval were also calculated to characterize early intraoperative variability. This approach allowed the model to capture not only the absolute physiologic state but also the deviation from each patient's own stable reference.

### Feature engineering and outcome definition

The primary outcome was intraoperative hypotension occurring within the subsequent 5 min, defined as MAP <65 mmHg at time (t + 5). For each eligible prediction time point, predictors were derived from a rolling 10-minute observation window consisting of observations at (t−10), (t−5), and (t).

Feature categories included: (1) current-value features (MAP_current, HR_current, SpO₂_current, EtCO₂_current); (2) lagged raw-value features (MAP_lag_5, MAP_lag_10); (3) baseline-relative features including absolute deviations (MAP_dev=MAP_t−MAP_baseline, HR_dev=HR_t−HR_baseline) and relative changes (relative_MAP_drop=(MAP_t−MAP_baseline)/MAP_baseline, relative_HR_drop=(HR_t−HR_baseline)/HR_baseline); (4) dynamic trend features (MAP_dev_slope, MAP_dev_change_5 m); (5) variability features (standard deviation of physiologic values across the current and preceding two 5-minute observations); (6) hypotensive burden features (MAP_lt70_count_10 m, MAP_lt70_exposure_10 m, MAP_lt65_count_10 m), also calculated across the current and preceding two 5-minute observations; and (7) contextual features (vasopressor exposure, elapsed anesthesia time, elapsed operative time, preincision_flag). Only time points with sufficient preceding observations to construct the complete rolling window were considered eligible for prediction.

### Missing data handling

Missing data were handled separately for preoperative static variables and intraoperative dynamic variables. Missing continuous preoperative variables were imputed using the median; missing categorical variables were imputed using the mode. For intraoperative variables, data were organized on a regular 5-minute time grid. Missing continuous values were imputed by linear interpolation when both preceding and subsequent measurements were available. Missing categorical or binary dynamic values were imputed using predefined rule-based filling. For missing values at the beginning or end of a sequence, forward or backward filling was used cautiously. In the final feature dataset, missingness was low overall; the only variables with non-zero missingness were smoking history and alcohol history, each with 50 missing observations (0.284%), and these were imputed using mode imputation. No interpolation was performed because the included intraoperative time-series data contained no missing values.

### Model development

Model development was performed using a fully nested case-grouped validation framework. Internal validation used an outer 5-fold stratified grouped cross-validation, while preprocessing, LASSO feature selection, and hyperparameter optimization were performed within an inner 5-fold stratified grouped cross-validation. The LASSO regularization parameter (C = 0.01, 0.1, 1.0, and 10.0) was optimized in the inner loop, and predictors with non-zero coefficients were retained. Four supervised classifiers (logistic regression, random forest, Extra Trees, and XGBoost) were subsequently trained and evaluated. All preprocessing steps were fitted exclusively within the training folds and applied unchanged to the corresponding held-out data. Decision thresholds were selected using the Youden index from grouped inner out-of-fold predictions and fixed before evaluation in the outer test folds. Performance was summarized from pooled outer-fold predictions, with 95% confidence intervals estimated by patient-level bootstrap resampling. SHAP analyses were performed only on held-out outer-fold data. Supplementary Material S2 provides the complete retained predictors and LR coefficients.

### Model performance evaluation

Discrimination was assessed using AUROC and AUPRC, with emphasis on AUPRC due to anticipated class imbalance. Threshold-dependent metrics included sensitivity, specificity, accuracy, and F1 score. Calibration was evaluated using Brier score, calibration slope, calibration intercept, and calibration plots. Clinical usefulness was assessed using decision curve analysis (DCA) (21). Discrimination and calibration of each model are summarized in Figure 3 Bootstrap resampling at the patient level was used to estimate 95% confidence intervals for major performance metrics.

### Primary analyses

The primary analyses evaluated model performance at both the window level and the patient/case level. Window-level performance assessed discrimination and calibration for individual 5-minute prediction windows, whereas patient-level performance summarized predictions across repeated windows within each surgical case, with case-level outcome status defined by the presence of at least one positive window and case-level prediction derived from the corresponding aggregated case-level model outputs.

### Secondary analyses

To further evaluate the robustness, generalizability, and clinical interpretability of the developed models beyond the primary analyses, several prespecified additional analyses were performed. First, sensitivity analyses were conducted under several complementary scenarios, including an incident-only analysis restricted to prediction windows with a current MAP ≥65 mmHg, extended-horizon analyses in which the outcome was redefined as hypotension occurring within the subsequent 10 or 15 min, and an alternative baseline definition, which defined the first 10–30 min of the anesthetic period as the baseline for feature construction. Second, comparator analyses were performed using reduced feature sets, including models based on current MAP alone, current MAP combined with lagged MAP values, current MAP combined with trend features and simple deterministic threshold rules based on the current MAP (<65, <68, <70, <72, and <75 mmHg) were also evaluated as clinically interpretable baseline comparators. Third, ablation analyses were conducted by excluding the current MAP value and, separately, excluding current hypotension-burden features to quantify their respective contributions to predictive performance. Fourth, subgroup analyses were performed across clinically relevant MAP strata to assess model discrimination under different hemodynamic conditions. Finally, external validation was performed by applying the finalized internally developed model artifacts, including the fixed decision thresholds, to an independent external cohort, with predictive performance evaluated at both the window and patient levels. For all primary and secondary analyses, predictive performance was estimated from pooled held-out outer-fold predictions generated within the fully nested case-grouped resampling framework. Ninety-five percent confidence intervals were obtained by nonparametric bootstrap resampling at the patient level.

### Model interpretability

For the logistic regression model, predictor effects were summarized using regression coefficients and odds ratios with 95% confidence intervals. For tree-based models, feature importance was evaluated using model-based importance measures and SHAP analysis (22). SHAP summary and dependence plots were used to characterize the relative contribution of each predictor to model-assigned risk and the pattern of model dependence, rather than to imply causal effects. To reduce optimistic bias, SHAP analyses were conducted within the nested grouped framework: for each outer fold, SHAP values were computed only on the corresponding held-out outer-fold data using models trained on the matched training folds, and values were then aggregated across held-out folds for the best-performing model. Model-based feature-importance rankings for tree models were interpreted as descriptive summaries and considered alongside SHAP results rather than as unbiased standalone evidence.

## Results

### Study population and cohort characteristics

A total of 702 patients from SHFU were included in the internal cohort, and 248 patients from FHXU comprised the external validation cohort. Because the analytic dataset was constructed on a regularized 5-minute intraoperative grid, these cohorts contributed 17,592 and 6,292 eligible prediction windows, respectively, of which 1,620 and 676 were positive by the final label definition. At the patient level, 419 internal patients and 155 external patients had at least one positive prediction window, while 415 and 155 patients, respectively, experienced at least one IOH episode, which was defined as each new onset of MAP <65 mmHg on the regularized 5-minute grid. A total of 1,035 IOH episodes were identified in the internal cohort and 393 in the external cohort. The median number of prediction windows per patient was 25 (IQR, 22–27) in the internal cohort and 22.0 (IQR, 20.0–25.0) in the external cohort. The median case duration was 125 min (IQR, 110–135) in the internal cohort and 128.0 min (IQR, 112.0–134.0) in the external cohort.

The detailed baseline characteristics of both cohorts are presented in Table 1. The internal and external cohorts showed broadly similar distributions across many demographic, comorbidity, laboratory, surgical, and medication variables, although differences were present for selected measures. The median age was 62.2 years (IQR, 54.1–72.2) in the internal cohort and 62.5 years (IQR, 53.8–73.3) in the external cohort. Females comprised 49.0% and 46.4% of the internal and external cohorts, respectively. Hypertension was the most prevalent comorbidity in both cohorts (24.8% vs. 28.2%). Emergency surgery accounted for 15.0% and 15.3% of cases in the internal and external cohorts, respectively. Preoperative vital signs and most laboratory variables were also similar between cohorts, although some differences were observed in selected measurements such as diastolic blood pressure, sodium, potassium, and glucose. The spaghetti plots of intraoperative physiological time-series variables (MAP, HR, SpO2, and EtCO2) shown in Figure 2 demonstrate substantial inter-patient variability in hemodynamic trajectories, supporting the need for individualized prediction approaches.

**Table 1:** Baseline characteristics of the study population.

Variables	Internal cohort (*n* = 702)	External cohort (*n* = 248)	Standardized Mean Differences
Demographics			
Age, years	62.2 [54.1–72.2]	62.5 [53.8–73.3]	−0.02
Female, *n* (%)	344 (49.0%)	115 (46.4%)	0.05
Body mass index, kg/m^2^	24.9 [22.5–27.5]	24.8 [22.5–27.6]	0.03
Smoking history, *n* (%)	95 (13.6%)	34 (13.7%)	−0.01
Alcohol history, *n* (%)	52 (7.4%)	19 (7.7%)	−0.01
Comorbidities			
Hypertension	174 (24.8%)	70 (28.2%)	0.08
Coronary artery disease	83 (11.8%)	35 (14.1%)	0.07
Heart failure	36 (5.1%)	11 (4.4%)	0.03
Arrhythmia history	48 (6.8%)	16 (6.5%)	0.02
Myocardial infarction history	24 (3.4%)	9 (3.6%)	0.01
Stroke history	34 (4.8%)	11 (4.4%)	0.02
Diabetes mellitus	94 (13.4%)	31 (12.5%)	0.03
Chronic kidney disease	72 (10.3%)	27 (10.9%)	0.02
Chronic respiratory disease	35 (5.0%)	15 (6.0%)	0.05
Malignancy	35 (5.0%)	14 (5.6%)	0.03
Preoperative vital signs			
Systolic blood pressure, mmHg	127 [116–139]	128 [115–138]	−0.06
Diastolic blood pressure, mmHg	74 [68–80]	72 [65–81]	0.19
Mean arterial pressure, mmHg	93 [86–98]	92 [85–99]	0.1
Heart rate, bpm	80 [72–89]	79 [71–87]	0.08
Laboratory measurements			
Hemoglobin, g/L	130.7 [119.9–142.2]	130.9 [118.5–142.8]	−0.01
Hematocrit	0.4 [0.4–0.5]	0.4 [0.4–0.5]	0
White blood cell count, ×10⁹/L	6.8 [5.6–8.2]	6.9 [5.4–8.4]	−0.05
Platelet count, ×10⁹/L	227.5 [190.3–264.1]	223.6 [187.8–263.3]	0.07
Creatinine, μmol/L	80.6 [62.9–100.1]	79.6 [63.2–99.8]	0.04
Blood urea nitrogen, mmol/L	5.7 [4.500–7.2]	5.8 [4.6–7.3]	−0.05
Sodium, mmol/L	140.2 [137.7–141.9]	139.6 [137.7–141.7]	0.2
Potassium, mmol/L	4.1 [3.9–4.4]	4.3 [3.8–4.5]	−0.44
Chloride, mmol/L	102.9 [100.6–105.4]	102.9 [101.1–105.5]	0
Calcium, mmol/L	2.4 [2.3–2.4]	2.4 [2.2–2.5]	0
Albumin, g/L	41.4 [38.3–44.1]	41.5 [38.1–44.5]	−0.02
Glucose, mmol/L	5.9 [5.1–7.0]	5.5 [5.2–7.3]	0.27
Prothrombin time, s	12.3 [11.4–13.1]	12.4 [11.4–13.2]	−0.08
INR	1.0 [1.0–1.1]	1.0 [0.9–1.3]	0
APTT, s	31.5 [28.6–34.3]	31.450 [28.8–34.2]	0.01
Surgical characteristics			
Emergency surgery	105 (15.0%)	38 (15.3%)	0.01
Surgery type			
Gastrointestinal	106 (15.1%)	28 (11.3%)	0.11
Orthopedic	97 (13.8%)	32 (12.9%)	0.03
Spine	87 (12.4%)	38 (15.3%)	0.08
Hepatobiliary	72 (10.3%)	27 (10.9%)	0.02
General surgery	64 (9.1%)	28 (11.3%)	0.07
Otorhinolaryngology	58 (8.3%)	26 (10.5%)	0.08
Thoracic	56 (8.0%)	22 (8.9%)	0.03
Orthopedic trauma	58 (8.3%)	18 (7.3%)	0.04
Gynecologic	52 (7.4%)	13 (5.2%)	0.09
Urologic	34 (4.8%)	10 (4.0%)	0.04
Endocrine	18 (2.6%)	6 (2.4%)	0.01
Preoperative medications			
Beta-blocker use	97 (13.8%)	43 (17.3%)	0.1
ACEI/ARB use	125 (17.8%)	43 (17.3%)	0.01
Diuretic use	57 (8.1%)	21 (8.5%)	0.01

Continuous variables: median [IQR]; categorical variables: *n* (%). ACEI, angiotensin-converting enzyme inhibitor; ARB, angiotensin receptor blocker; APTT, activated partial thromboplastin time; INR, international normalized ratio.

**Figure 2:**
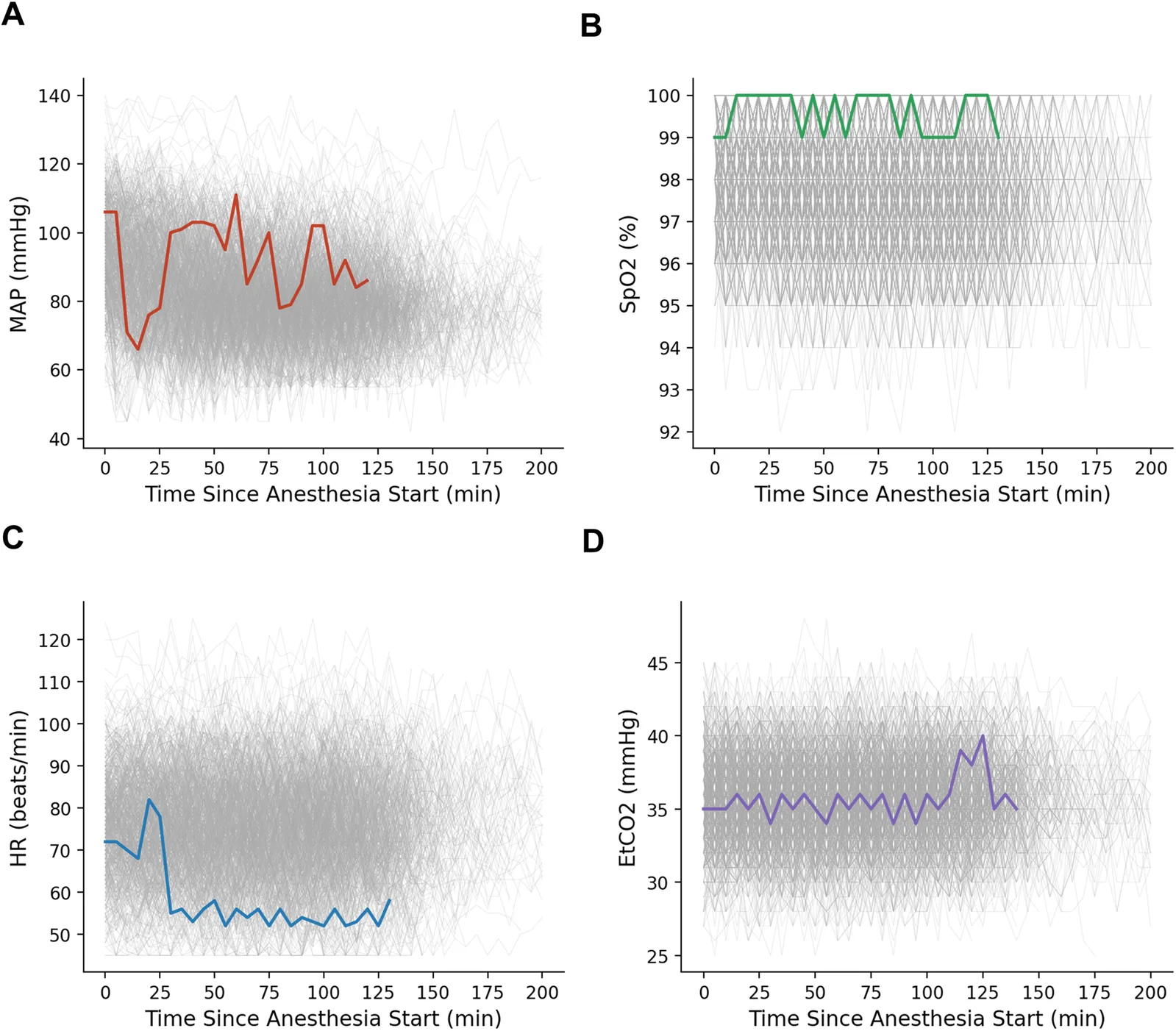
Intraoperative physiological time-series trajectories of internal cohort. Spaghetti plots of mean arterial pressure **(A)**, SpO₂ **(B)**, heart rate **(C)**, and EtCO₂ **(D)** Gray lines represent individual patients; colored lines highlight selected patient trajectories over time.

**Figure 3:**
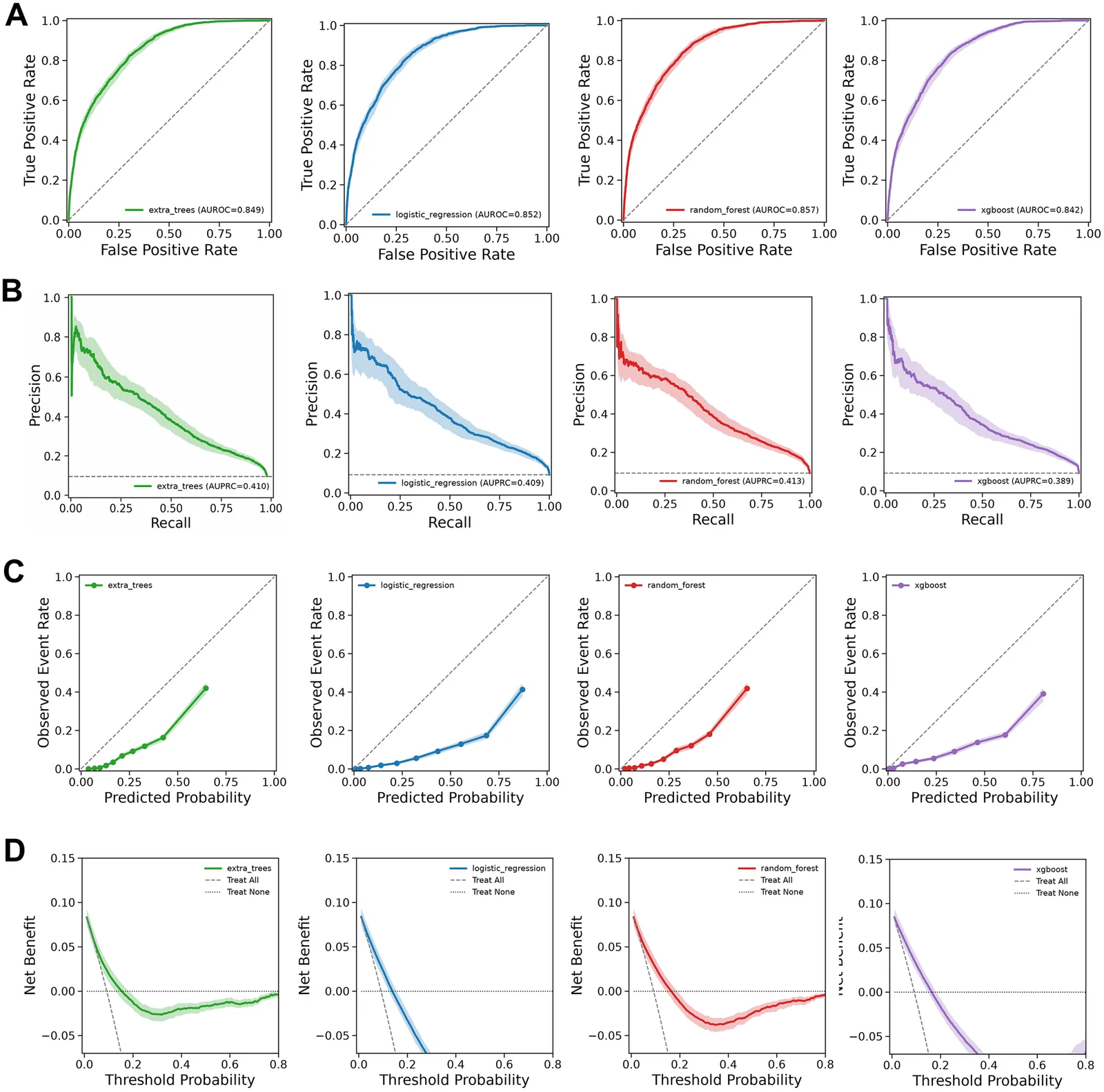
Predictive performance of machine learning models based on internal cohort. **(A)** ROC curves, **(B)** precision–recall curves, **(C)** calibration plots, and **(D)** decision curve analysis comparing Extra Trees, Random Forest, XGBoost, and logistic regression for intraoperative hypotension prediction in the SHFU internal validation dataset. Performance was evaluated using 702 cases and 17,592 eligible 5-minute prediction windows, including 1,620 positive windows (9.2%).

### Model development and feature selection

LASSO logistic regression with cross-validated penalty parameter selection identified a parsimonious set of predictors with non-zero coefficients. Retained features included MAP_current, MAP_lag_5, MAP_lag_10, MAP baseline deviation features, recent hypotensive burden features (MAP_lt70_count_10 m, MAP_lt65_exposure_10 m), HR_current, elapsed anesthesia time, and selected preoperative variables. The LASSO procedure effectively eliminated redundant or non-informative predictors, reducing model dimensionality while preserving predictive capacity.

### Primary analyses

The predictive performance of the four candidate models in the primary fully nested case-grouped internal validation is summarized in Tables 2, 3. At the window level (Table 2), all models demonstrated similar discriminatory ability, with AUROC values ranging from 0.842 to 0.857. Random forest achieved the highest AUROC (0.857, 95% CI 0.841–0.868) and the highest AUPRC (0.413, 95% CI 0.367–0.452). Logistic regression and extra trees also showed competitive performance, with AUROCs of 0.852 (95% CI 0.838–0.865) and 0.849 (95% CI 0.833–0.860), respectively. XGBoost demonstrated comparable discrimination, with an AUROC of 0.842 (95% CI 0.825–0.854). Model calibration varied across algorithms. Extra trees achieved the lowest Brier score (0.093, 95% CI 0.088–0.098), indicating the best overall probabilistic accuracy, whereas logistic regression exhibited the calibration slope closest to the ideal value of 1 (0.898, 95% CI 0.853–0.944). The remaining models also demonstrated acceptable calibration, with calibration slopes ranging from 0.871 to 1.358. Logistic recalibration produced calibration curves that more closely approximated the ideal line across all four models, indicating improved agreement between predicted and observed probabilities (Supplementary Figure S2).

**Table 2:** Window-level predictive performance of candidate machine learning models for intraoperative hypotension in the internal validation cohort.

Model	AUROC (95% CI)	AUPRC (95% CI)	Brier Score (95% CI)	Sensitivity (95% CI)	Specificity (95% CI)	Accuracy (95% CI)	F1 Score (95% CI)	Calibration Intercept (95% CI)	Calibration Slope (95% CI)	Threshold
random_forest	0.857 (0.841–0.868)	0.413 (0.367–0.452)	0.095 (0.090–0.101)	0.805 (0.778–0.825)	0.730 (0.708–0.750)	0.737 (0.718–0.754)	0.360 (0.337–0.381)	−1.233 (-1.309−1.180)	1.358 (1.286–1.410)	0.310
logistic_regression	0.852 (0.838–0.865)	0.409 (0.352–0.455)	0.158 (0.147–0.169)	0.875 (0.854–0.895)	0.653 (0.624–0.679)	0.673 (0.650–0.696)	0.330 (0.308–0.352)	-2.226 (-2.290−2.162)	0.898 (0.853–0.944)	0.470
extra_trees	0.849 (0.833–0.860)	0.410 (0.361–0.451)	0.093 (0.088–0.098)	0.828 (0.804–0.847)	0.687 (0.664–0.709)	0.700 (0.681–0.719)	0.337 (0.313–0.357)	-1.160 (-1.224–1.097)	1.351 (1.291–1.396)	0.270
xgboost	0.842 (0.825–0.854)	0.389 (0.332–0.431)	0.126 (0.118–0.136)	0.862 (0.831–0.880)	0.650 (0.621–0.676)	0.670 (0.645–0.692)	0.324 (0.301–0.346)	-1.788 (-1.865–1.712)	0.871 (0.791–0.923)	0.320

AUROC, area under the receiver operating characteristic curve; AUPRC, area under the precision-recall curve; CI, confidence interval.

**Table 3:** Patient-level predictive performance of candidate machine learning models in the internal validation cohort.

Model	AUROC (95% CI)	AUPRC (95% CI)	Sensitivity (95% CI)	Specificity (95% CI)	Accuracy (95% CI)	F1 Score (95% CI)	Calibration Intercept (95% CI)	Calibration Slope (95% CI)	Mean alerts per case-hour (95% CI)
xgboost	0.921 (0.908–0.936)	0.942 (0.928–0.956)	0.962 (0.948–0.973)	0.604 (0.566–0.649)	0.818 (0.796–0.838)	0.863 (0.846–0.880)	0.105 (-0.056–0.267)	1.703 (1.524–1.863)	4.286 (6.170–7.382)
random_forest	0.956 (0.948–0.964)	0.970 (0.962–0.977)	0.998 (0.996–1.000)	0.664 (0.619–0.709)	0.863 (0.840–0.885)	0.897 (0.878–0.913)	1.336 (1.100–1.511)	3.597 (3.241–3.750)	3.832 (5.587–6.638)
logistic_regression	0.945 (0.936–0.957)	0.962 (0.953–0.972)	0.962 (0.949–0.975)	0.668 (0.625–0.704)	0.843 (0.826–0.866)	0.880 (0.864–0.898)	-0.330 (-0.501–0.137)	2.158 (1.954–2.381)	4.256 (6.179–7.214)
extra_trees	0.947 (0.936–0.956)	0.963 (0.953–0.971)	0.995 (0.992–1.000)	0.640 (0.598–0.677)	0.852 (0.835–0.868)	0.889 (0.873–0.901)	1.593 (1.372–1.804)	3.053 (2.779–3.228)	4.278 (6.218–7.320)

AUROC, area under the receiver operating characteristic curve; AUPRC, area under the precision-recall curve; CI, confidence interval.

Threshold-dependent performance revealed distinct operating characteristics. Logistic regression achieved the highest sensitivity (0.875, 95% CI 0.854–0.895), followed by XGBoost (0.862, 95% CI 0.831–0.880), whereas random forest provided the highest specificity (0.730, 95% CI 0.708–0.750) and the highest overall accuracy (0.737, 95% CI 0.718–0.754). Extra trees demonstrated intermediate performance, with a sensitivity of 0.828 (95% CI 0.804–0.847) and specificity of 0.687 (95% CI 0.664–0.709). F1 scores were broadly comparable across models, ranging from 0.324 for XGBoost to 0.360 for random forest, reflecting the persistent challenge posed by class imbalance in intraoperative hypotension prediction. Across prespecified probability thresholds of 0.2, 0.4, 0.6, and 0.8, all four models showed the expected threshold-dependent tradeoff between sensitivity and specificity, whereas AUROC, AUPRC, and Brier score remained essentially unchanged (Supplementary Table S1).

ROC curves demonstrated consistent discrimination across all four models, with Random Forest exhibiting marginally superior AUROC values. Precision-recall curves highlighted the anticipated class imbalance, with all models substantially outperforming baseline prevalence. Calibration curves demonstrated acceptable agreement between predicted probabilities and observed outcomes across all models. Decision curve analysis confirmed that all models provided net benefit over both the “treat-all” and “treat-none” strategies across clinically relevant threshold probability ranges.

At the patient level (Table 3), discrimination improved substantially for all models, with AUROC values ranging from 0.921 to 0.956 and AUPRC values from 0.942 to 0.970. Random forest achieved the highest AUROC (0.956, 95% CI 0.948–0.964) and the highest AUPRC (0.970, 95% CI 0.962–0.977). Logistic regression and extra trees also demonstrated excellent discrimination, with AUROCs of 0.945 and 0.947, respectively, whereas XGBoost achieved an AUROC of 0.921 and an AUPRC of 0.942. Random forest achieved the highest sensitivity (0.998), specificity (0.664), and overall accuracy (0.863). Mean alert frequency remained below 4.3 alerts per case-hour for all models, with random forest generating the fewest alerts (3.832 per case-hour). Although calibration intercepts and slopes varied among algorithms, all models maintained excellent discrimination and clinically acceptable operating characteristics at the patient level.

### Secondary analyses

Sensitivity analyses demonstrated that model performance remained consistent across alternative analytic scenarios (Supplementary Table S2). In the incident-only analysis restricted to windows with current MAP ≥65 mmHg, random forest achieved the highest AUROC (0.827, 95% CI 0.815–0.842). For the 10-minute prediction horizon, XGBoost showed the best discrimination (AUROC 0.847, 95% CI 0.834–0.858), whereas random forest performed best at the 15-minute horizon (AUROC 0.840, 95% CI 0.826–0.852). Using an alternative baseline defined from the first 10–30 min after anesthesia induction produced similar results, with XGBoost achieving an AUROC of 0.856 (95% CI 0.841–0.866).

Comparator analyses are summarized in Supplementary Table S3. The full multivariable model outperformed all simplified MAP-based comparators. The best-performing current-MAP-only model achieved an AUROC of 0.823 (95% CI 0.808–0.835), compared with 0.857 (95% CI 0.841–0.868) for the primary model. Incorporating recent MAP measurements improved discrimination (best AUROC 0.845, 95% CI 0.830–0.856), although performance remained below that of the full model. Similar performance was observed when current MAP was combined with simple trend features (best AUROC 0.845, 95% CI 0.829–0.855). Deterministic rule-based comparators using fixed current MAP thresholds (<65 to <75 mmHg) demonstrated the expected trade-off between sensitivity (0.354–0.726) and specificity (0.936–0.764), but none provided the balanced predictive performance achieved by the proposed model.

Ablation analyses (Supplementary Table S4) showed that excluding MAP_current alone had little effect on discrimination, with the best-performing model achieving an AUROC of 0.858 (95% CI 0.842–0.868). In contrast, excluding hypotension-burden features reduced performance, with the highest AUROC decreasing to 0.845 (95% CI 0.829–0.856). However, the MAP_current ablation should be interpreted as testing dependence on the single raw current MAP variable rather than on all current MAP-related information, because several retained features still incorporated current or near-current MAP measurements.

Stratified analyses according to current MAP are presented in Supplementary Table S5. For the random forest model, AUROC was 0.814 for windows with current MAP ≥75 mmHg, compared with 0.686 for MAP 70–74 mmHg and 0.661 for MAP 65–69 mmHg. The corresponding AUPRC values were 0.124, 0.255, and 0.373, respectively.

External Validation Performance.

The predictive performance of the four candidate models in the independent external validation cohort is summarized in Supplementary Table S6. At the window level, all models maintained good discrimination, with AUROC values ranging from 0.852 to 0.894. Random forest achieved the best overall performance, yielding the highest AUROC (0.894, 95% CI 0.875–0.907), highest AUPRC (0.540, 95% CI 0.457–0.596), lowest Brier score (0.092, 95% CI 0.083–0.102), highest sensitivity (0.871, 95% CI 0.835–0.898), and highest F1 score (0.441, 95% CI 0.399–0.476). XGBoost achieved the highest specificity (0.773, 95% CI 0.736–0.805) and accuracy (0.774, 95% CI 0.743–0.801). Calibration was acceptable across all models, with XGBoost exhibiting the calibration slope closest to the ideal value of 1 (0.993, 95% CI 0.901–1.081). Calibration curves demonstrated systematic underestimation of event risk for several models before recalibration, whereas logistic recalibration substantially improved agreement between predicted and observed probabilities in the external cohort (Supplementary Figure S3). The decision-curve analysis demonstrated a similar pattern to that observed in the internal validation cohort, supporting the consistency of the model's potential clinical utility across cohorts (Supplementary Figure S4).

At the patient level, discrimination was strong across all models, with AUROC values ranging from 0.946 to 0.971 and AUPRC values ranging from 0.966 to 0.982. Random forest achieved the highest AUROC (0.971, 95% CI 0.950–0.987), highest AUPRC (0.982, 95% CI 0.969–0.993), and highest sensitivity (0.981, 95% CI 0.958–0.997). Specificity was highest for XGBoost (0.772, 95% CI 0.703–0.849). Extra trees achieved the highest accuracy (0.924, 95% CI 0.899–0.952) and F1 score (0.898, 95% CI 0.866–0.935). XGBoost generated the lowest alert burden, with a mean of 3.488 alerts per case-hour (95% CI 3.028–4.004), and all models generated fewer than 4.1 alerts per case-hour.

### Feature importance and model interpretability

SHAP analysis identified consistent patterns of feature importance across the four evaluated models (Figure 4). In Extra Trees (Figure 4A), recent hypotensive burden (MAP Lt70 Exposure 10 m) was the dominant contributor. XGBoost (Figure 4B) likewise identified recent hypotensive burden (MAP Lt70 Exposure 10 m) as the most influential predictor, followed by current MAP. Current MAP ranked highest in both Random Forest (Figure 4C) and Logistic Regression (Figure 4D). Despite these differences, all models consistently prioritized MAP-derived features, including current MAP, recent hypotensive burden (MAP Lt70 Exposure 10 m and MAP Lt65 Count 10 m), recent MAP measurements (MAP Lag5 and MAP Lag10), relative MAP reduction, and MAP variability. Elapsed anesthesia or operative time and current vasopressor exposure were also among the most influential predictors. SHAP summary plots further demonstrated that lower current MAP, greater recent hypotensive burden, larger relative MAP reductions, and current vasopressor exposure were generally associated with increased predicted risk of intraoperative hypotension.

**Figure 4:**
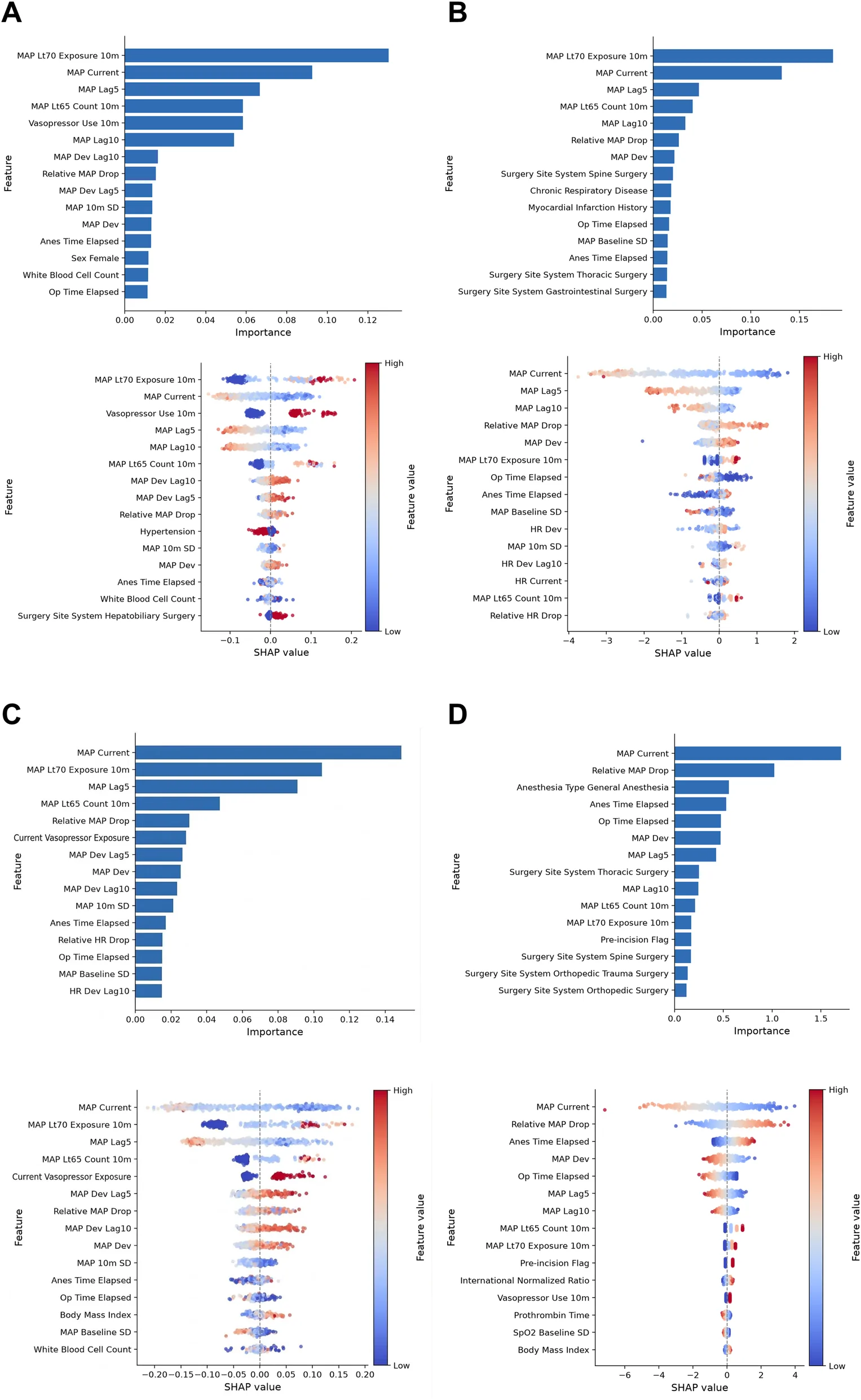
Feature importance and SHAP-based interpretability based on internal cohort. Global feature importance rankings and SHAP summary plots illustrating the contribution of each predictor to model predictions across four modeling approaches. **(A)** Extra Trees, **(B)** XGBoost, **(C)** Random Forest, and **(D)** Logistic Regression.

### Relationship between predicted risk and MAP dynamics

A strong inverse correlation was observed between observed MAP and predicted hypotension probability (R = −0.807, *P* < 0.001), confirming the physiological coherence of the model predictions (Figure 5A). Concordance analysis between temporal changes in MAP (*Δ*MAP) and corresponding changes in predicted probability (*Δ*Predicted probability) showed that 67.3% of consecutive transitions exhibited directionally concordant behavior, including 34.6% in Quadrant II and 32.7% in Quadrant IV (Figure 5B). The remaining 32.7% of transitions were directionally discordant, comprising 15.7% in Quadrant I and 17.0% in Quadrant III. These results demonstrate that changes in predicted risk were generally aligned with the direction of temporal MAP changes across consecutive intraoperative observations.

**Figure 5:**
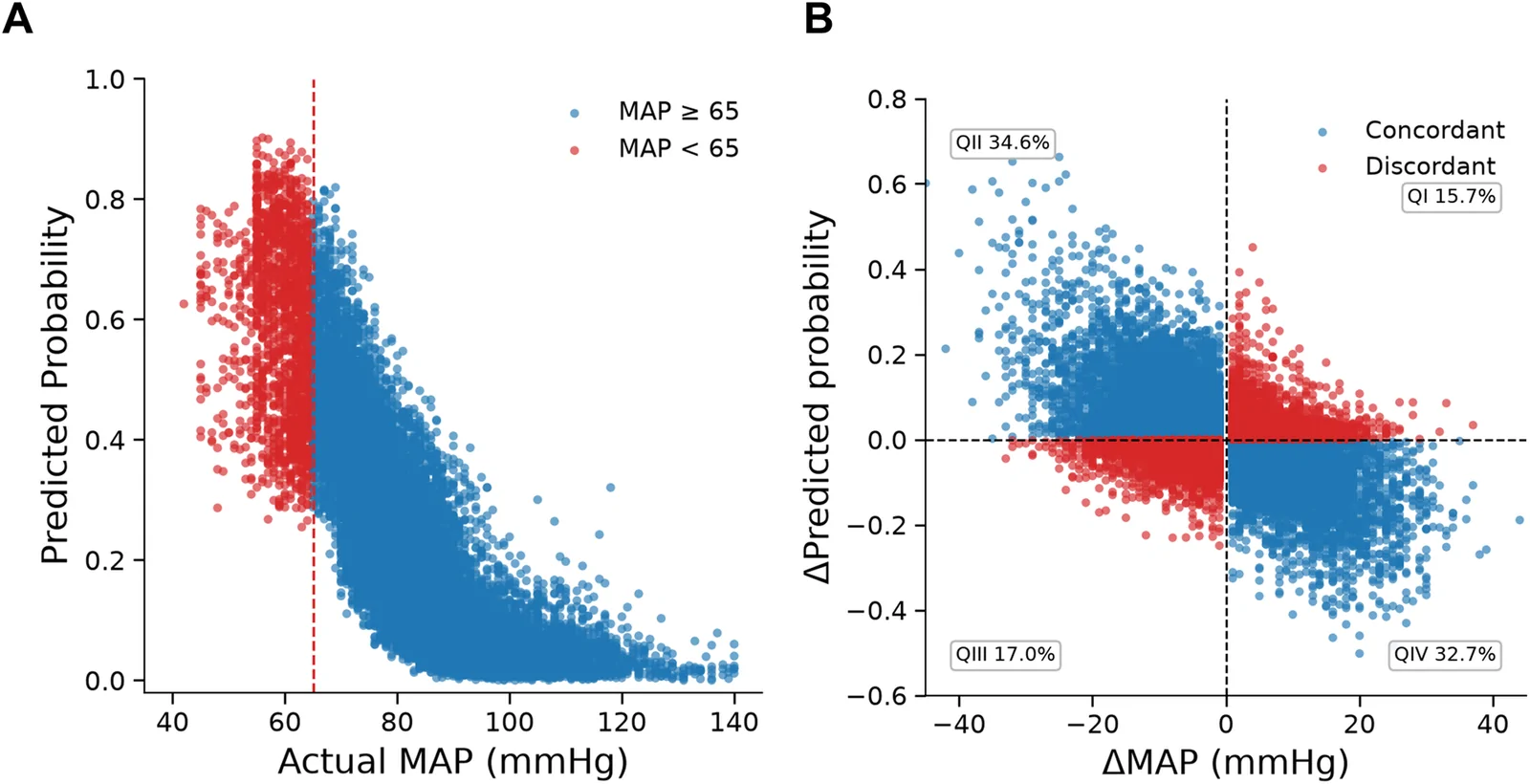
Relationship between predicted hypotension risk and MAP dynamics (random forest model) based on internal cohort. **(A)** Inverse correlation between predicted probability and actual value of predicted MAP (R = −0.807, *P* < 0.001). Each point represents a time point across all patients. **(B)** Concordance analysis shows that 67.3% of transitions align with hemodynamic directionality. Each point represents the change between two consecutive time points. QI denotes increasing MAP with increasing predicted risk, QII denotes decreasing MAP with increasing predicted risk, QIII denotes decreasing MAP with decreasing predicted risk, and QIV denotes increasing MAP with decreasing predicted risk. Blue points indicate concordant transitions (QII and QIV), whereas red points indicate discordant transitions (QI and QIII).

### Patient-level dynamic prediction

Representative patient-level trajectories illustrating the temporal concordance between MAP and model-predicted risk are presented. Patient A exhibited gradual hemodynamic deterioration with a corresponding escalation in predicted risk preceding the onset of intraoperative hypotension. Three IOH episodes occurred during the procedure, all of which were successfully identified by the model, although two additional false-positive high-risk alerts were also observed (Figure 6A). Patient B demonstrated stable intraoperative hemodynamics with persistently low predicted probabilities throughout the procedure, and the model did not predict any intraoperative hypotension episodes during surgery (Figure 6B). These case-based visualizations exemplify the model's capacity for dynamic risk assessment.

**Figure 6:**
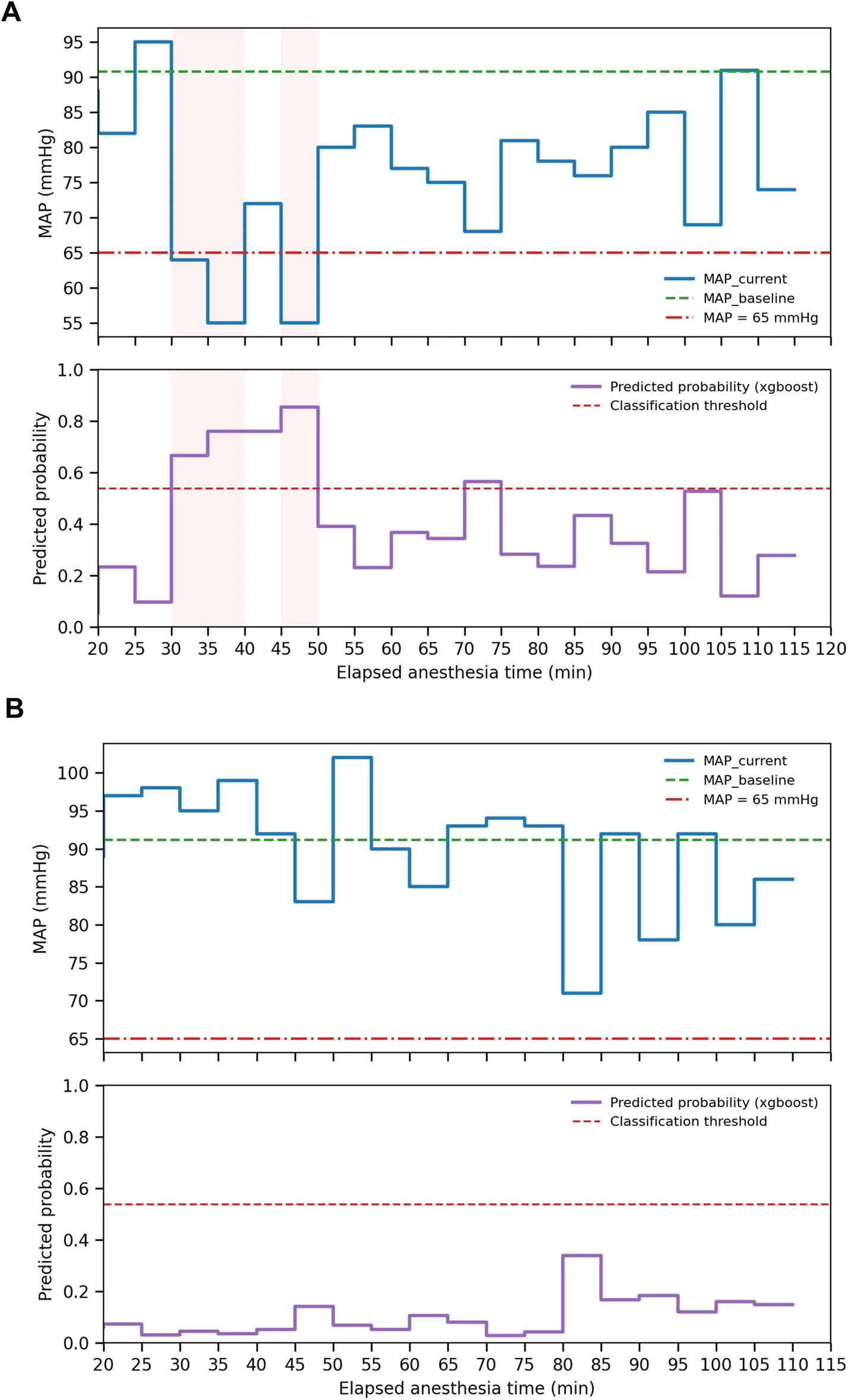
Representative patient-level trajectories with predicted risk from internal cohort. Two intraoperative cases showing temporal alignment between mean arterial pressure trends and model-predicted hypotension risk across surgical time points. **(A)** Patient A and **(B)** Patient B.

## Discussion

This multicenter retrospective study demonstrates that a dynamic machine-learning model integrating routinely collected perioperative data with an individualized intraoperative baseline and comprehensive time-series feature engineering can predict intraoperative hypotension in a 5-minute horizon with strong discrimination, acceptable calibration, and favorable decision-curve performance. In internal validation, Random Forest achieved the highest AUROC (0.857), and its performance remained robust in the independent external validation cohort (AUROC 0.894), confirming transportability across independent hospital settings. The integration of patient-specific baselines, SHAP-based interpretability, and decision curve analysis distinguishes this work from existing literature and provides practical insights for clinical translation.

The predictive performance achieved in this study is consistent with, and in several respects comparable to, previously reported IOH prediction models. The pooled AUROC of 0.90 reported in the systematic review by Shirmohamadi et al. for HPI across 22 studies (12), and the median AUROC of 0.912 reported by Koh et al. in a scoping review of 26 studies (13), serve as important benchmarks. Our external validation AUROC of 0.894 falls within this range, despite using only routinely available noninvasive monitoring variables rather than high-fidelity arterial waveform data. This finding has important practical implications: a significant proportion of surgical patients are not monitored with invasive arterial catheters, and a prediction model capable of leveraging standard monitoring data extends the population eligible for IOH early warning. The Transformer-based model developed by Zhu et al. achieved 5-minute prediction AUROC of 0.904 using 319,699 cases (16), further reinforcing the upper performance boundary achievable with deep learning on large-scale datasets. However, the relative simplicity and interpretability of our tree-based framework may confer implementation advantages in resource-constrained clinical environments.

A distinguishing methodological strength of this study is the incorporation of individualized intraoperative baselines. By defining each patient's early stable physiologic reference during the initial 20 min post-induction, we capture not merely the absolute MAP value, but also the magnitude of deviation from that patient's own norm. This approach aligns with the growing recognition that “one-size-fits-all” blood pressure targets may be suboptimal and that personalized hemodynamic management may reduce organ injury (19, 20). Schnetz and colleagues developed a model predicting patient-specific MAP targets associated with the lowest IOH risk, incorporating age, sex, and comorbidities (23). Similarly, personalized perioperative blood pressure management strategies are increasingly advocated, with emerging evidence suggesting that patient-specific targets may improve outcomes compared with fixed population-level thresholds (24).

The comprehensive feature engineering framework employed in this study represents another notable advancement. While previous models have utilized various time-series representations, our systematic approach encompassing current-value features, lagged trajectory features, baseline-relative deviations, dynamic trend quantifications, variability metrics, and recent hypotensive burden features captures complementary dimensions of hemodynamic risk. This multi-faceted approach aligns with prior work demonstrating the value of systematic feature engineering for hypotension prediction (14) and recent innovations employing trend and seasonal decomposition for dynamic sequence modeling (17). The inclusion of hypotensive burden features is particularly noteworthy, as cumulative exposure to MAP below specific thresholds has been independently linked to AKI and myocardial injury in multiple studies (3, 4, 6, 25). By encoding recent hypotensive burden into the prediction framework, our model implicitly captures the temporal accumulation of hemodynamic stress.

In this study, the Youden index was used to determine the classification threshold during model development because it provides a standardized criterion for comparing candidate models under balanced operating conditions. However, this threshold should not be interpreted as the definitive operating point for clinical deployment. In practice, the optimal threshold should be tailored to the intended clinical application and the acceptable balance between missed hypotensive events and false alarms. To facilitate clinical implementation, we additionally report model performance across multiple clinically relevant decision thresholds, allowing institutions to select an operating point appropriate for their workflow and available resources.

Although current MAP was the most influential predictor, comparator and ablation analyses demonstrated that incorporating individualized baseline characteristics, temporal hemodynamic dynamics, and recent hypotensive burden consistently improved discrimination over simplified MAP-based models. However, much of the predictive performance remained driven by recent MAP trajectory and hypotensive burden. Accordingly, the proposed framework should be interpreted as a short-horizon dynamic risk model anchored primarily in recent MAP dynamics rather than an independent physiologic forecasting model. Nevertheless, this retrospective study did not evaluate clinically relevant alarm-performance measures, such as first-alert lead time, alert burden, or episode-level sensitivity, and these should be assessed prospectively. Although external validation demonstrated consistent predictive performance, further prospective workflow validation is required before routine clinical implementation.

The strong inverse correlation between MAP and predicted probability (R = −0.807, *P* < 0.001) merits careful interpretation. While this correlation confirms the physiological coherence of model predictions, it also raises the question recently emphasized in critiques of HPI (11, 26) of whether ML-based prediction models offer incremental value beyond simple MAP threshold monitoring. Several considerations support the added value of our approach. First, the model incorporates not only current MAP but also trajectory features, baseline deviations, and hypotensive burden, providing a richer risk characterization than absolute MAP alone. Second, the concordance analysis demonstrates that the model captures the directionality of hemodynamic change in 67.3% of transitions, but importantly, the 32.7% discordant transitions may represent clinically informative scenarios where predicted risk diverges from absolute MAP potentially identifying impending decompensation before it is fully manifest in the MAP signal. Third, the decision curve analysis confirms net benefit across a range of threshold probabilities, indicating greater net benefit than simple MAP-based alarming on decision curve analysis. Nevertheless, future prospective studies should directly compare ML-guided hemodynamic management with MAP-threshold-based protocols to rigorously establish incremental benefit.

The model interpretability analysis via SHAP provides clinically actionable insights. The consistent identification of MAP_current, MAP baseline deviation, recent hypotensive burden, and elapsed anesthesia time as top predictors aligns with established physiological understanding of hypotension mechanisms (7, 27). Longer anesthesia duration reflects cumulative effects of anesthetic agents, fluid shifts, and progressive autonomic dysregulation. The contribution of preoperative variables such as age and hypertension history, while modest, is consistent with known risk factors for intraoperative hemodynamic instability (2, 5). By quantifying the relative importance of these predictors across models, SHAP analysis provides transparency that may facilitate clinician trust and adoption a critical consideration for risk stratification framework (28).

### Novel contributions

Several aspects of our methodology represent advances over prior work. First, the incorporation of individualized intraoperative baselines is a key innovation. Schnetz and colleagues demonstrated that intraoperative hypotension risk is patient-dependent and that personalized MAP targets can be predicted preoperatively (23); our approach extends this concept by computing dynamic, intraoperative baselines that adapt to each patient's evolving physiology. This contrasts with the Hypotension Prediction Index and similar algorithms that rely on population-derived waveform features without explicit patient-specific referencing (8, 9). Second, our comprehensive feature engineering framework encompassing current values, lagged measurements, baseline-relative deviations, dynamic trends, variability, hypotensive burden, and contextual variables provides a more complete representation of hemodynamic state than prior studies relying primarily on waveform-derived features (8, 14). Third, the LASSO-based feature selection approach balances predictive performance with model parsimony, reducing the risk of overfitting and enhancing clinical interpretability. This is particularly important given the relatively small sample size of our external validation cohort (*n* = 248) and the known tendency of complex machine-learning models to overfit when predictor-to-event ratios are unfavorable. Fourth, our comprehensive evaluation framework assessing discrimination, calibration, and interpretability addresses the growing recognition that reporting standards for machine-learning prediction studies require demonstration of calibration and clinical usefulness beyond AUROC alone (21, 22).

### Limitations

This study has several limitations. First, the retrospective design inherently limits causal inference; prospective interventional trials are required to determine whether model-guided hemodynamic management reduces IOH duration and, more importantly, postoperative organ injury. Recent trials of HPI-guided management have yielded mixed results regarding clinical outcomes (10, 29). Second, while the external validation cohort was from an independent hospital, both centers were tertiary-care institutions, potentially limiting generalizability to community or ambulatory surgery settings where IOH incidence patterns may differ (2). Third, the 5-minute prediction horizon, while clinically actionable, is shorter than the 15-minute horizon demonstrated by HPI (8, 9); extending the prediction window while maintaining accuracy represents an important direction for future work. Fourth, the study did not incorporate intraoperative medication administration data beyond vasopressor exposure (e.g., anesthetic depth, fluid administration rates), which may provide additional predictive value. Fifth, the class imbalance inherent in IOH prediction hypotensive events represent a minority of all intraoperative time points necessitates continued attention to precision-recall optimization and threshold calibration. Sixth, although the present study evaluated window-level and case-level performance, episode-level performance requires further prospective validation. Seventh, the exclusion of short procedures, cases with persistent early hypotension, and cases with severe data-quality problems may have removed clinically challenging situations in which early warning could still be relevant.

### Future directions

Future research directions include: (1) prospective randomized evaluation of model-guided vs. standard hemodynamic management, including workflow integration, clinician response, and alert burden; (2) integration of additional intraoperative data streams including processed EEG and noninvasive cardiac output monitoring; (3) multicenter validation in diverse surgical populations and practice settings, including assessment of algorithmic fairness across clinically relevant patient subgroups; and (4) investigation of whether individualized baseline integration meaningfully improves prediction over population-level approaches through dedicated comparative studies.

## Conclusions

This study demonstrates that a dynamic machine-learning framework leveraging routinely collected perioperative data, individualized intraoperative baselines, and systematic time-series feature engineering can predict intraoperative hypotension within a 5-minute horizon with strong discrimination, acceptable calibration, and favorable decision-curve performance. The model's external validation performance (Random Forest AUROC 0.894) and interpretability via SHAP analysis support the proposed framework as a candidate short-horizon risk stratification model. Prospective workflow validation is required before routine clinical implementation.

## Data Availability

The datasets presented in this article are not readily available because The datasets generated and analyzed during the current study are not publicly available due to institutional data governance policies and patient privacy regulations but are available from the corresponding author on reasonable request, subject to institutional approval and data sharing agreements.. Requests to access the datasets should be directed tot;Lijun Yang, yy00633@126.com.

## Supplementary material

Supplementary Figure S1

Study population selection flowchart. Flowchart showing inclusion and exclusion criteria applied to the external cohort (FHXU).

Supplementary Figure S2

Internal calibration performance of the four prediction models. Calibration plots for the four candidate models in the internal validation cohort. For each model, the left panel shows the original calibration of predicted probabilities, whereas the right panel shows calibration after logistic recalibration. The dashed gray diagonal represents the ideal calibration line. Blue (left) and red (right) curves indicate the smoothed calibration relationship between predicted probabilities and observed event rates, and dots represent deciles of predicted risk. Calibration was assessed using pooled held-out predictions obtained from the nested grouped cross-validation procedure. The recalibrated intercept and slope are displayed in the corresponding right panels. (A) Extra Trees, (B) XGBoost, (C) Random Forest, and (D) Logistic Regression.

Supplementary Figure S3

External calibration performance of the four prediction models. Calibration plots for the four candidate models in the independent external validation cohort. For each model, the left panel shows the original calibration of predicted probabilities, whereas the right panel shows calibration after logistic recalibration. The dashed gray diagonal represents the ideal calibration line. Blue (left) and red (right) curves indicate the smoothed calibration relationship between predicted probabilities and observed event rates, and dots represent deciles of predicted risk. Calibration performance was evaluated using predictions generated in the external validation cohort. The recalibrated intercept and slope are displayed in the corresponding right panels. (A) Extra Trees, (B) XGBoost, (C) Random Forest, and (D) Logistic Regression.

Supplementary Figure S4

Decision curve analysis of the four prediction models in the external validation cohort. Decision curve analysis (DCA) was performed using pooled out-of-fold predictions from the grouped nested cross-validation. Panels show the net benefit of (A) Extra Trees, (B) Logistic Regression, (C) Random Forest, and (D) XGBoost across a range of threshold probabilities.
