# Supplementary material for: Dynamic machine-learning model for early prediction of intraoperative hypotension using 5-minute perioperative time-series data: a multicenter retrospective study
Source: Front Med (Lausanne). 2026 Sep 30;13:1884174. doi: 10.3389/fmed.2026.1884174 (PMC13661343; doi:10.3389/fmed.2026.1884174)
Supplement: Supplementary file 1 [file Supplementaryfile1.docx]

**Supplementary Material 1****. Full Analytic Pipeline**

Step 1. Cohort construction

1. Identify eligible surgical cases from the internal cohort.

2. Exclude cases failing case-level eligibility criteria.

3. Exclude cases with persistent hypotension during the first 20 minutes after induction, defined as four consecutive 5-minute MAP values <65 mmHg.

4. Construct the external validation cohort separately using the same feature-construction logic.

Step 2. Time-grid regularization

1. Align intraoperative observations to a regularized 5-minute grid.

2. Define each eligible prediction time point as a grid time t with sufficient prior observations.

3. Require a complete 10-minute observation window so that predictors can be derived from (t−10), (t−5), and t.

Step 3. Outcome labeling

1. In the primary analysis, assign the binary label at time t according to whether MAP at the next scheduled charted time point, (t+5), is <65 mmHg.

2. For sensitivity analyses with extended horizons, redefine the outcome using the corresponding alternative horizon definition.

3. Store all labels on the regularized 5-minute grid rather than using continuously sampled values between charted time points.

Step 4. Feature construction

1. Derive contemporaneous physiologic predictors from time t.

2. Derive lagged and short-term trend features from (t−10) and (t−5).

3. Derive recent hypotension-burden features.

4. Derive vasopressor and elapsed-time features.

5. Derive individualized baseline-relative features using the early anesthetic period (0–20 minutes after anesthesia initiation).

Step 5. Define analytic structure

1. Treat each 5-minute prediction window as the primary analytic unit.

2. Retain case_id as the grouping variable.

3. Enforce grouped resampling so that all windows from the same surgical case remain in the same fold.

4. In the implementation, case_id was the grouping unit used for all grouped resampling, threshold selection, and bootstrap procedures.

5. The window-level label remained the dependent variable, whereas stratification for grouped resampling was performed at the case level using the case-level maximum of the binary outcome to preserve event balance across folds.

Step 6. Build preprocessing pipeline

1. Within each training partition, separate predictors into numeric and categorical variables.

2. Median-impute numeric predictors.

3. Standardize numeric predictors.

4. Most-frequent-impute categorical predictors.

5. One-hot encode categorical predictors.

Step 7. Add feature selection

1. Apply LASSO logistic regression after preprocessing within the same pipeline.

2. Tune the LASSO regularization parameter within the inner grouped cross-validation loop.

3. Retain predictors with non-zero coefficients.

4. If no coefficient is retained, keep the predictor with the largest absolute coefficient.

5. LASSO-based feature selection was implemented as a custom transformer using LogisticRegression(penalty='l1', solver='liblinear', class_weight='balanced', max_iter=2000).

6. The LASSO penalty-strength grid was C = [0.01, 0.1, 1.0, 10.0], tuned within the same inner grouped cross-validation loop as the downstream model hyperparameters.

7. Predictors were retained if the absolute fitted coefficient exceeded 1×10^-8. 8. The full training pipeline order was: preprocessing → LASSO feature selection → downstream classifier.

Step 8. Specify candidate models

1. Logistic regression was implemented as LogisticRegression(max_iter=2000, class_weight='balanced', solver='liblinear') with hyperparameter grid model__C = [0.1, 1.0, 10.0].

2. Random forest was implemented as RandomForestClassifier(random_state=42, n_jobs=-1, class_weight='balanced') with grid model__n_estimators = [200, 400], model__max_depth = [None, 8, 16], and model__min_samples_leaf = [1, 5].

3. Extra trees was implemented as ExtraTreesClassifier(random_state=42, n_jobs=-1, class_weight='balanced') with grid model__n_estimators = [200, 400], model__max_depth = [None, 8, 16], and model__min_samples_leaf = [1, 5].

4. XGBoost was implemented as XGBClassifier(random_state=42, n_estimators=300, eval_metric='logloss', tree_method='hist', n_jobs=-1, scale_pos_weight = n_negative / n_positive within the corresponding outer-training data) with grid model__max_depth = [3, 5], model__learning_rate = [0.03, 0.1], and model__subsample = [0.8, 1.0].

Step 9. Nested grouped internal validation

1. Perform outer 5-fold stratified grouped cross-validation using case_id for internal performance evaluation.

2. For each outer training set:

a. perform inner 5-fold stratified grouped cross-validation using case_id,

b. fit preprocessing,

c. fit LASSO feature selection,

d. perform model-specific hyperparameter tuning by grid search.

Step 10. Hyperparameter selection

1. For each candidate model, evaluate the hyperparameter grid within the inner grouped cross-validation procedure.

2. Select the best hyperparameter combination according to the prespecified inner-loop performance criterion.

3. Reconstruct the best pipeline template for that outer training set.

Step 11. Threshold selection

1. Generate grouped inner out-of-fold predicted probabilities within the outer training set using the selected pipeline.

2. Evaluate candidate thresholds across a prespecified threshold grid.

3. Select the threshold using the Youden index.

4. Fix this threshold for the corresponding outer held-out fold.

Step 12. Held-out outer-fold evaluation

1. Refit the selected pipeline on the full outer training set.

2. Predict probabilities on the held-out outer fold only.

3. Apply the fixed threshold selected from the inner out-of-fold predictions.

4. Store held-out predictions, fold-specific metrics, and selected-feature counts.

Step 13. Internal pooled performance summary

1. Pool predictions across all held-out outer folds.

2. Compute window-level performance metrics from pooled held-out predictions.

3. Report discrimination, calibration, and classification metrics.

Step 14. Bootstrap confidence intervals

1. Sample cases with replacement at the case_id level.

2. Retain all windows belonging to each sampled case.

3. Recompute performance metrics on each bootstrap sample.

4. Use the bootstrap distribution to estimate 95% confidence intervals.

Step 15. Case-level and episode-level summaries

1. Aggregate repeated window-level predictions within each surgical case to generate case-level summaries.

2. Define an IOH episode as each new onset of MAP <65 mmHg on the regularized 5-minute grid, with contiguous subsequent time points below 65 mmHg treated as the same episode.

3. Quantify whether alerts preceded episode onset and summarize alert lead times.

Step 16. Additional and sensitivity analyses

1. Incident-only analysis: windows with current MAP ≥65 mmHg

2. Longer-horizon analyses: 10-minute and 15-minute prediction settings

3. Comparator analyses: reduced predictor sets

4. Ablation analyses: exclusion of selected predictor groups

5. MAP-band analyses: performance within clinically relevant MAP strata

6. Baseline sensitivity analysis: alternative baseline definition

Step 17. Train final internal model artifacts

1. Using the full internal cohort, refit each final model pipeline with:

a. preprocessing,

b. LASSO-based feature selection,

c. selected hyperparameters.

2. Save the fitted pipeline, selected threshold, and feature-selection metadata for each model.

Step 18. External validation

1. Apply the same feature-construction logic to the external cohort.

2. Load the saved final internal model artifacts.

3. Apply each artifact unchanged to the external cohort.

4. Generate external predicted probabilities and classification outputs using the fixed internal threshold.

5. Report external performance metrics separately from internal results.

Step 19. Interpretability analysis

1. For logistic regression, summarize predictor effects using coefficients and odds ratios with 95% confidence intervals.

2. For tree-based models, summarize predictor importance using model-based importance measures and SHAP.

3. Within the nested grouped workflow, compute SHAP values only on held-out outer-fold data using models trained on the matched outer-training folds.

4. Aggregate SHAP values across held-out outer folds for the best-performing model.

5. Interpret SHAP as describing contributions to model-assigned risk and dependence structure rather than causal effects.

Step 20. Reproducibility reporting

1. Document preprocessing order,

2. grouped resampling structure,

3. random-seed control,

4. hyperparameter search strategy,

5. feature-selection logic,

6. threshold-selection procedure,

7. internal and external validation workflow,

8. interpretability workflow.

9. software environment: Python 3.11.15, numpy 2.4.4, pandas 3.0.2, scikit-learn 1.8.0, joblib 1.5.3, and xgboost 3.2.0
