# Supplementary material for: Dynamic machine-learning model for early prediction of intraoperative hypotension using 5-minute perioperative time-series data: a multicenter retrospective study
Source: Front Med (Lausanne). 2026 Sep 30;13:1884174. doi: 10.3389/fmed.2026.1884174 (PMC13661343; doi:10.3389/fmed.2026.1884174)
Supplement: Supplementary file 2 [file Table1.docx]

**Supplementary Table 1. Window-level performance of four prediction models across probability thresholds**

| **Model** | **Threshold** | **AUROC**  **(95% CI)** | **AUPRC**  **(95% CI)** | **Brier Score**  **(95% CI)** | **Sensitivity**  **(95% CI)** | **Specificity**  **(95% CI)** | **Accuracy**  **(95% CI)** | **F1 Score**  **(95% CI)** |
| --- | --- | --- | --- | --- | --- | --- | --- | --- |
| logistic_regression | 0.2 | 0.852 (0.836-0.870) | 0.409 (0.366-0.465) | 0.158 (0.148-0.168) | 0.963 (0.954-0.974) | 0.466 (0.438-0.489) | 0.512 (0.489-0.531) | 0.267 (0.251-0.288) |
| random_forest |  | 0.857 (0.842-0.870) | 0.413 (0.373-0.454) | 0.095 (0.089-0.102) | 0.933 (0.916-0.944) | 0.567 (0.533-0.595) | 0.600 (0.572-0.626) | 0.301 (0.278-0.321) |
| extra_trees |  | 0.849 (0.835-0.862) | 0.410 (0.346-0.455) | 0.093 (0.088-0.099) | 0.917 (0.900-0.930) | 0.572 (0.548-0.604) | 0.604 (0.584-0.632) | 0.299 (0.281-0.317) |
| xgboost |  | 0.842 (0.827-0.857) | 0.389 (0.346-0.440) | 0.126 (0.117-0.135) | 0.915 (0.897-0.934) | 0.556 (0.524-0.580) | 0.589 (0.562-0.608) | 0.291 (0.269-0.308) |
| logistic_regression | 0.4 | 0.852 (0.835-0.865) | 0.409 (0.354-0.479) | 0.158 (0.146-0.169) | 0.862 (0.836-0.879) | 0.671 (0.647-0.695) | 0.689 (0.669-0.710) | 0.338 (0.316-0.360) |
| random_forest |  | 0.857 (0.843-0.870) | 0.413 (0.363-0.453) | 0.095 (0.089-0.102) | 0.660 (0.625-0.696) | 0.841 (0.825-0.856) | 0.824 (0.809-0.839) | 0.409 (0.381-0.436) |
| extra_trees |  | 0.849 (0.837-0.865) | 0.410 (0.367-0.466) | 0.093 (0.088-0.098) | 0.584 (0.543-0.624) | 0.877 (0.865-0.888) | 0.850 (0.839-0.861) | 0.418 (0.386-0.451) |
| xgboost |  | 0.842 (0.825-0.857) | 0.389 (0.343-0.436) | 0.126 (0.118-0.134) | 0.765 (0.733-0.797) | 0.748 (0.727-0.766) | 0.749 (0.730-0.765) | 0.360 (0.334-0.385) |
| logistic_regression | 0.6 | 0.852 (0.834-0.865) | 0.409 (0.358-0.461) | 0.158 (0.148-0.167) | 0.662 (0.613-0.700) | 0.834 (0.819-0.851) | 0.818 (0.804-0.833) | 0.401 (0.364-0.430) |
| random_forest |  | 0.857 (0.843-0.871) | 0.413 (0.365-0.457) | 0.095 (0.090-0.101) | 0.362 (0.314-0.404) | 0.964 (0.958-0.970) | 0.908 (0.899-0.916) | 0.421 (0.373-0.462) |
| extra_trees |  | 0.849 (0.835-0.862) | 0.410 (0.361-0.464) | 0.093 (0.087-0.099) | 0.323 (0.279-0.371) | 0.969 (0.962-0.973) | 0.909 (0.899-0.918) | 0.396 (0.347-0.444) |
| xgboost |  | 0.842 (0.826-0.856) | 0.389 (0.346-0.438) | 0.126 (0.117-0.133) | 0.530 (0.484-0.575) | 0.886 (0.876-0.900) | 0.853 (0.843-0.867) | 0.400 (0.367-0.431) |
| logistic_regression | 0.8 | 0.852 (0.838-0.868) | 0.409 (0.359-0.459) | 0.158 (0.147-0.168) | 0.402 (0.352-0.466) | 0.950 (0.939-0.958) | 0.899 (0.888-0.909) | 0.424 (0.383-0.472) |
| random_forest |  | 0.857 (0.844-0.872) | 0.413 (0.361-0.464) | 0.095 (0.089-0.102) | 0.049 (0.031-0.067) | 0.998 (0.997-0.999) | 0.910 (0.902-0.921) | 0.091 (0.059-0.122) |
| extra_trees |  | 0.849 (0.835-0.861) | 0.410 (0.363-0.465) | 0.093 (0.088-0.098) | 0.083 (0.048-0.123) | 0.997 (0.996-0.998) | 0.913 (0.905-0.920) | 0.149 (0.090-0.212) |
| xgboost |  | 0.842 (0.824-0.853) | 0.389 (0.330-0.428) | 0.126 (0.118-0.135) | 0.273 (0.221-0.308) | 0.974 (0.970-0.979) | 0.910 (0.899-0.918) | 0.358 (0.303-0.395) |

**Supplementary Table 2. Sensitivity analyses of model performance under alternative analytic scenarios**

**Scenario A. Incident-only analysis**

| **Model** | **AUROC**  **(95% CI)** | **AUPRC**  **(95% CI)** | **Brier Score**  **(95% CI)** | **Sensitivity**  **(95% CI)** | **Specificity**  **(95% CI)** | **Accuracy**  **(95% CI)** | **F1 Score**  **(95% CI)** | **Calibration Intercept (95% CI)** | **Calibration Slope**  **(95% CI)** |
| --- | --- | --- | --- | --- | --- | --- | --- | --- | --- |
| random_forest | 0.827 (0.815-0.842) | 0.239 (0.217-0.266) | 0.062 (0.057-0.068) | 0.782 (0.753-0.814) | 0.715 (0.691-0.741) | 0.720 (0.699-0.743) | 0.267 (0.253-0.286) | -0.730 (-0.822--0.609) | 1.159 (1.094-1.235) |
| xgboost | 0.825 (0.811-0.839) | 0.235 (0.218-0.260) | 0.160 (0.149-0.171) | 0.858 (0.832-0.883) | 0.640 (0.614-0.669) | 0.654 (0.630-0.680) | 0.245 (0.232-0.262) | -2.427 (-2.496--2.349) | 0.942 (0.879-1.019) |
| logistic_regression | 0.824 (0.811-0.839) | 0.234 (0.214-0.258) | 0.180 (0.166-0.193) | 0.842 (0.811-0.868) | 0.660 (0.629-0.689) | 0.672 (0.645-0.697) | 0.251 (0.237-0.271) | -2.611 (-2.686--2.523) | 0.831 (0.783-0.891) |
| extra_trees | 0.811 (0.798-0.825) | 0.241 (0.215-0.271) | 0.111 (0.104-0.119) | 0.511 (0.470-0.550) | 0.852 (0.835-0.868) | 0.830 (0.814-0.845) | 0.282 (0.263-0.304) | -1.902 (-1.982--1.828) | 1.005 (0.950-1.067) |

**Scenario B. Prediction horizon = 10 min**

| **Model** | **AUROC**  **(95% CI)** | **AUPRC**  **(95% CI)** | **Brier Score**  **(95% CI)** | **Sensitivity**  **(95% CI)** | **Specificity**  **(95% CI)** | **Accuracy**  **(95% CI)** | **F1 Score**  **(95% CI)** | **Calibration Intercept (95% CI)** | **Calibration Slope**  **(95% CI)** |
| --- | --- | --- | --- | --- | --- | --- | --- | --- | --- |
| xgboost | 0.847 (0.834-0.858) | 0.510 (0.464-0.546) | 0.156 (0.147-0.166) | 0.800 (0.772-0.829) | 0.730 (0.703-0.750) | 0.740 (0.721-0.757) | 0.480 (0.457-0.501) | -1.597 (-1.656--1.526) | 1.062 (1.005-1.116) |
| random_forest | 0.845 (0.831-0.856) | 0.512 (0.470-0.548) | 0.105 (0.099-0.112) | 0.845 (0.826-0.865) | 0.685 (0.656-0.707) | 0.709 (0.690-0.727) | 0.466 (0.445-0.486) | -0.510 (-0.575--0.447) | 1.253 (1.192-1.316) |
| extra_trees | 0.840 (0.827-0.853) | 0.508 (0.466-0.544) | 0.127 (0.120-0.134) | 0.784 (0.760-0.808) | 0.720 (0.696-0.743) | 0.730 (0.713-0.747) | 0.465 (0.445-0.487) | -1.078 (-1.129--1.017) | 1.303 (1.245-1.359) |
| logistic_regression | 0.840 (0.826-0.852) | 0.500 (0.455-0.538) | 0.165 (0.156-0.177) | 0.741 (0.709-0.771) | 0.762 (0.738-0.783) | 0.759 (0.741-0.775) | 0.480 (0.455-0.503) | -1.690 (-1.753--1.625) | 0.906 (0.858-0.964) |

**Scenario C. Prediction horizon = 15 min**

| **Model** | **AUROC**  **(95% CI)** | **AUPRC**  **(95% CI)** | **Brier Score**  **(95% CI)** | **Sensitivity**  **(95% CI)** | **Specificity**  **(95% CI)** | **Accuracy**  **(95% CI)** | **F1 Score**  **(95% CI)** | **Calibration Intercept (95% CI)** | **Calibration Slope**  **(95% CI)** |
| --- | --- | --- | --- | --- | --- | --- | --- | --- | --- |
| random_forest | 0.840 (0.826-0.852) | 0.569 (0.530-0.602) | 0.124 (0.117-0.132) | 0.835 (0.812-0.860) | 0.693 (0.661-0.718) | 0.721 (0.700-0.739) | 0.541 (0.519-0.561) | -0.436 (-0.499--0.368) | 1.253 (1.192-1.321) |
| xgboost | 0.839 (0.824-0.852) | 0.561 (0.520-0.595) | 0.158 (0.149-0.168) | 0.865 (0.846-0.885) | 0.657 (0.628-0.685) | 0.698 (0.679-0.716) | 0.530 (0.511-0.551) | -1.238 (-1.305--1.170) | 1.052 (0.996-1.114) |
| logistic_regression | 0.834 (0.820-0.847) | 0.554 (0.516-0.591) | 0.168 (0.159-0.179) | 0.803 (0.777-0.827) | 0.706 (0.676-0.731) | 0.725 (0.705-0.742) | 0.534 (0.508-0.557) | -1.367 (-1.423--1.301) | 0.924 (0.874-0.986) |
| extra_trees | 0.833 (0.819-0.846) | 0.551 (0.510-0.584) | 0.145 (0.137-0.153) | 0.770 (0.744-0.800) | 0.736 (0.712-0.758) | 0.743 (0.726-0.760) | 0.541 (0.519-0.564) | -0.973 (-1.022--0.908) | 1.200 (1.136-1.261) |

**Scenario D. Alternative baseline definition**

| **Model** | **AUROC**  **(95% CI)** | **AUPRC**  **(95% CI)** | **Brier Score**  **(95% CI)** | **Sensitivity**  **(95% CI)** | **Specificity**  **(95% CI)** | **Accuracy**  **(95% CI)** | **F1 Score**  **(95% CI)** | **Calibration Intercept (95% CI)** | **Calibration Slope**  **(95% CI)** |
| --- | --- | --- | --- | --- | --- | --- | --- | --- | --- |
| xgboost | 0.856 (0.841-0.866) | 0.420 (0.360-0.461) | 0.149 (0.140-0.159) | 0.824 (0.793-0.844) | 0.709 (0.682-0.733) | 0.719 (0.697-0.740) | 0.351 (0.329-0.372) | -2.120 (-2.188--2.059) | 1.064 (1.004-1.109) |
| random_forest | 0.854 (0.837-0.865) | 0.411 (0.361-0.454) | 0.075 (0.070-0.080) | 0.806 (0.773-0.830) | 0.726 (0.702-0.747) | 0.734 (0.714-0.750) | 0.358 (0.332-0.380) | -0.690 (-0.778--0.610) | 1.169 (1.108-1.228) |
| logistic_regression | 0.852 (0.836-0.865) | 0.414 (0.359-0.458) | 0.160 (0.149-0.172) | 0.793 (0.764-0.818) | 0.732 (0.708-0.755) | 0.738 (0.717-0.758) | 0.358 (0.332-0.382) | -2.250 (-2.319--2.188) | 0.922 (0.872-0.964) |
| extra_trees | 0.852 (0.835-0.863) | 0.422 (0.370-0.462) | 0.114 (0.108-0.121) | 0.810 (0.784-0.832) | 0.716 (0.693-0.736) | 0.724 (0.706-0.742) | 0.351 (0.326-0.373) | -1.713 (-1.770--1.662) | 1.038 (0.985-1.078) |

**Supplementary Table 3. Comparator analyses of model performance using reduced MAP-based feature sets**

**A. current MAP only**

| **Model** | **AUROC**  **(95% CI)** | **AUPRC**  **(95% CI)** | **Brier Score**  **(95% CI)** | **Sensitivity**  **(95% CI)** | **Specificity**  **(95% CI)** | **Accuracy**  **(95% CI)** | **F1 Score**  **(95% CI)** | **Calibration Intercept (95% CI)** | **Calibration Slope**  **(95% CI)** |
| --- | --- | --- | --- | --- | --- | --- | --- | --- | --- |
| logistic_regression | 0.823 (0.808-0.835) | 0.315 (0.274-0.347) | 0.177 (0.169-0.186) | 0.759 (0.732-0.779) | 0.733 (0.717-0.749) | 0.735 (0.722-0.748) | 0.345 (0.322-0.367) | -2.278 (-2.351--2.208) | 0.939 (0.871-0.989) |
| xgboost | 0.821 (0.805-0.832) | 0.333 (0.288-0.367) | 0.176 (0.169-0.185) | 0.741 (0.711-0.760) | 0.748 (0.732-0.763) | 0.747 (0.733-0.760) | 0.351 (0.326-0.371) | -2.241 (-2.314--2.165) | 0.983 (0.901-1.045) |
| extra_trees | 0.821 (0.805-0.833) | 0.340 (0.291-0.377) | 0.183 (0.178-0.189) | 0.761 (0.734-0.782) | 0.729 (0.713-0.746) | 0.732 (0.718-0.746) | 0.343 (0.320-0.365) | -2.275 (-2.349--2.202) | 2.044 (1.900-2.169) |
| random_forest | 0.816 (0.800-0.828) | 0.333 (0.291-0.369) | 0.158 (0.151-0.165) | 0.720 (0.691-0.741) | 0.764 (0.750-0.780) | 0.760 (0.747-0.774) | 0.356 (0.333-0.379) | -2.069 (-2.137--1.993) | 1.053 (0.973-1.117) |

**B. current MAP + lagged MAP**

| **Model** | **AUROC**  **(95% CI)** | **AUPRC**  **(95% CI)** | **Brier Score**  **(95% CI)** | **Sensitivity**  **(95% CI)** | **Specificity**  **(95% CI)** | **Accuracy**  **(95% CI)** | **F1 Score**  **(95% CI)** | **Calibration Intercept (95% CI)** | **Calibration Slope**  **(95% CI)** |
| --- | --- | --- | --- | --- | --- | --- | --- | --- | --- |
| extra_trees | 0.845 (0.830-0.856) | 0.410 (0.361-0.445) | 0.138 (0.130-0.145) | 0.752 (0.723-0.773) | 0.771 (0.753-0.790) | 0.769 (0.754-0.785) | 0.375 (0.349-0.399) | -1.960 (-2.013--1.904) | 1.133 (1.071-1.173) |
| xgboost | 0.844 (0.828-0.855) | 0.408 (0.362-0.446) | 0.162 (0.153-0.170) | 0.783 (0.754-0.803) | 0.724 (0.703-0.745) | 0.729 (0.711-0.747) | 0.347 (0.324-0.370) | -2.202 (-2.253--2.135) | 0.987 (0.927-1.027) |
| random_forest | 0.842 (0.826-0.853) | 0.401 (0.355-0.437) | 0.137 (0.129-0.143) | 0.773 (0.745-0.794) | 0.736 (0.716-0.757) | 0.739 (0.722-0.757) | 0.353 (0.331-0.375) | -1.927 (-1.984--1.871) | 0.971 (0.911-1.009) |
| logistic_regression | 0.841 (0.825-0.853) | 0.368 (0.327-0.404) | 0.170 (0.161-0.179) | 0.786 (0.759-0.806) | 0.730 (0.709-0.751) | 0.735 (0.717-0.752) | 0.353 (0.331-0.374) | -2.290 (-2.345--2.229) | 0.977 (0.914-1.020) |

**C. current MAP + trend features**

| **Model** | **AUROC**  **(95% CI)** | **AUPRC**  **(95% CI)** | **Brier Score**  **(95% CI)** | **Sensitivity**  **(95% CI)** | **Specificity**  **(95% CI)** | **Accuracy**  **(95% CI)** | **F1 Score**  **(95% CI)** | **Calibration Intercept (95% CI)** | **Calibration Slope**  **(95% CI)** |
| --- | --- | --- | --- | --- | --- | --- | --- | --- | --- |
| extra_trees | 0.845 (0.829-0.855) | 0.408 (0.360-0.444) | 0.139 (0.131-0.146) | 0.750 (0.717-0.771) | 0.769 (0.751-0.788) | 0.767 (0.752-0.784) | 0.372 (0.345-0.395) | -1.970 (-2.023--1.913) | 1.141 (1.077-1.182) |
| xgboost | 0.844 (0.828-0.855) | 0.406 (0.360-0.444) | 0.161 (0.152-0.170) | 0.780 (0.753-0.801) | 0.725 (0.705-0.746) | 0.730 (0.712-0.748) | 0.348 (0.325-0.370) | -2.198 (-2.250--2.131) | 0.985 (0.926-1.026) |
| random_forest | 0.843 (0.827-0.853) | 0.401 (0.356-0.436) | 0.136 (0.128-0.143) | 0.777 (0.750-0.797) | 0.738 (0.718-0.759) | 0.742 (0.724-0.760) | 0.357 (0.334-0.380) | -1.928 (-1.984--1.870) | 0.985 (0.926-1.024) |
| logistic_regression | 0.841 (0.825-0.853) | 0.368 (0.327-0.404) | 0.170 (0.161-0.179) | 0.786 (0.759-0.806) | 0.730 (0.709-0.751) | 0.735 (0.717-0.752) | 0.353 (0.331-0.374) | -2.290 (-2.345--2.229) | 0.976 (0.914-1.019) |

**D. simple rule-based threshold**

| **Rule** | **AUROC-like score (95% CI)** | **Sensitivity (95% CI)** | **Specificity (95% CI)** | **Accuracy (95% CI)** |
| --- | --- | --- | --- | --- |
| MAP current <65 mmHg | 0.823 (0.807–0.838) | 0.354 (0.313–0.398) | 0.936 (0.930–0.942) | 0.883 (0.873–0.892) |
| MAP current <68 mmHg | 0.823 (0.807–0.838) | 0.470 (0.431–0.512) | 0.902 (0.894–0.909) | 0.862 (0.852–0.872) |
| MAP current <70 mmHg | 0.823 (0.807–0.838) | 0.557 (0.517–0.597) | 0.869 (0.858–0.877) | 0.840 (0.829–0.850) |
| MAP current <72 mmHg | 0.823 (0.807–0.838) | 0.631 (0.596–0.664) | 0.832 (0.820–0.843) | 0.813 (0.801–0.825) |
| MAP current <75 mmHg | 0.823 (0.807–0.838) | 0.726 (0.693–0.754) | 0.764 (0.748–0.777) | 0.760 (0.746–0.773) |

**Supplementary Table 4. Ablation analyses of model performance after excluding key MAP-related features**

**A. Excluding hypotension-burden features**

| **Model** | **AUROC**  **(95% CI)** | **AUPRC**  **(95% CI)** | **Brier Score**  **(95% CI)** | **Sensitivity**  **(95% CI)** | **Specificity**  **(95% CI)** | **Accuracy**  **(95% CI)** | **F1 Score**  **(95% CI)** | **Calibration Intercept (95% CI)** | **Calibration Slope**  **(95% CI)** |
| --- | --- | --- | --- | --- | --- | --- | --- | --- | --- |
| xgboost | 0.845 (0.829-0.856) | 0.357 (0.308-0.398) | 0.154 (0.145-0.165) | 0.836 (0.808-0.861) | 0.695 (0.664-0.717) | 0.708 (0.682-0.727) | 0.345 (0.321-0.367) | -2.098 (-2.179--2.020) | 1.019 (0.950-1.086) |
| logistic_regression | 0.843 (0.827-0.856) | 0.373 (0.318-0.417) | 0.165 (0.156-0.177) | 0.852 (0.829-0.869) | 0.673 (0.645-0.697) | 0.690 (0.666-0.709) | 0.336 (0.313-0.358) | -2.247 (-2.315--2.175) | 0.929 (0.872-0.971) |
| random_forest | 0.840 (0.824-0.851) | 0.359 (0.299-0.405) | 0.080 (0.075-0.086) | 0.809 (0.773-0.835) | 0.713 (0.685-0.733) | 0.722 (0.698-0.741) | 0.349 (0.323-0.372) | -0.618 (-0.716--0.513) | 1.315 (1.227-1.391) |
| extra_trees | 0.836 (0.821-0.847) | 0.362 (0.307-0.414) | 0.108 (0.102-0.115) | 0.786 (0.748-0.811) | 0.721 (0.696-0.744) | 0.727 (0.705-0.747) | 0.346 (0.321-0.368) | -1.280 (-1.349--1.207) | 1.524 (1.415-1.610) |

**B. Excluding current MAP**

| **Model** | **AUROC**  **(95% CI)** | **AUPRC**  **(95% CI)** | **Brier Score**  **(95% CI)** | **Sensitivity**  **(95% CI)** | **Specificity**  **(95% CI)** | **Accuracy**  **(95% CI)** | **F1 Score**  **(95% CI)** | **Calibration Intercept (95% CI)** | **Calibration Slope**  **(95% CI)** |
| --- | --- | --- | --- | --- | --- | --- | --- | --- | --- |
| random_forest | 0.858 (0.842-0.868) | 0.421 (0.371-0.463) | 0.073 (0.069-0.079) | 0.827 (0.800-0.850) | 0.711 (0.685-0.732) | 0.721 (0.700-0.739) | 0.353 (0.329-0.374) | -0.553 (-0.632--0.488) | 1.276 (1.217-1.326) |
| xgboost | 0.857 (0.842-0.867) | 0.412 (0.362-0.452) | 0.149 (0.140-0.158) | 0.849 (0.818-0.869) | 0.690 (0.662-0.714) | 0.705 (0.681-0.725) | 0.346 (0.324-0.367) | -2.124 (-2.184--2.059) | 1.084 (1.019-1.130) |
| logistic_regression | 0.852 (0.837-0.865) | 0.411 (0.355-0.456) | 0.159 (0.149-0.171) | 0.794 (0.762-0.820) | 0.734 (0.708-0.756) | 0.739 (0.718-0.758) | 0.359 (0.335-0.384) | -2.247 (-2.309--2.184) | 0.924 (0.879-0.963) |
| extra_trees | 0.851 (0.835-0.862) | 0.405 (0.353-0.445) | 0.108 (0.101-0.115) | 0.693 (0.658-0.725) | 0.812 (0.796-0.829) | 0.801 (0.787-0.816) | 0.391 (0.361-0.412) | -1.587 (-1.635--1.525) | 1.120 (1.073-1.158) |

**Supplementary Table 5. Subgroup analyses of model performance across clinically relevant MAP strata**

**A. Band of MAP ≥75 mmHg**

| **Model** | **AUROC**  **(95% CI)** | **AUPRC**  **(95% CI)** | **Brier Score**  **(95% CI)** | **Sensitivity**  **(95% CI)** | **Specificity**  **(95% CI)** | **Accuracy**  **(95% CI)** | **F1 Score**  **(95% CI)** | **Calibration Intercept (95% CI)** | **Calibration Slope**  **(95% CI)** |
| --- | --- | --- | --- | --- | --- | --- | --- | --- | --- |
| extra_trees | 0.801 (0.779-0.817) | 0.110 (0.094-0.129) | 0.052 (0.048-0.056) | 0.507 (0.463-0.553) | 0.838 (0.820-0.854) | 0.827 (0.808-0.843) | 0.170 (0.149-0.189) | -1.249 (-1.425--1.061) | 1.407 (1.279-1.523) |
| logistic_regression | 0.811 (0.790-0.832) | 0.117 (0.100-0.142) | 0.087 (0.080-0.095) | 0.610 (0.563-0.653) | 0.817 (0.797-0.834) | 0.810 (0.790-0.827) | 0.184 (0.163-0.206) | -2.394 (-2.531--2.273) | 0.853 (0.774-0.962) |
| random_forest | 0.814 (0.791-0.831) | 0.124 (0.108-0.143) | 0.050 (0.045-0.054) | 0.392 (0.352-0.439) | 0.898 (0.884-0.911) | 0.881 (0.865-0.894) | 0.187 (0.163-0.212) | -1.233 (-1.432--1.044) | 1.359 (1.204-1.496) |
| xgboost | 0.809 (0.789-0.826) | 0.120 (0.101-0.138) | 0.066 (0.060-0.073) | 0.613 (0.564-0.655) | 0.803 (0.779-0.824) | 0.797 (0.773-0.817) | 0.175 (0.156-0.189) | -2.094 (-2.236--1.980) | 0.764 (0.699-0.825) |

**B. Band of MAP 70–74 mmHg**

| **Model** | **AUROC**  **(95% CI)** | **AUPRC**  **(95% CI)** | **Brier Score**  **(95% CI)** | **Sensitivity**  **(95% CI)** | **Specificity**  **(95% CI)** | **Accuracy**  **(95% CI)** | **F1 Score**  **(95% CI)** | **Calibration Intercept (95% CI)** | **Calibration Slope**  **(95% CI)** |
| --- | --- | --- | --- | --- | --- | --- | --- | --- | --- |
| extra_trees | 0.660 (0.629-0.694) | 0.250 (0.216-0.305) | 0.146 (0.138-0.153) | 0.803 (0.754-0.854) | 0.412 (0.386-0.445) | 0.467 (0.443-0.493) | 0.298 (0.269-0.326) | -1.026 (-1.176--0.803) | 1.061 (0.884-1.298) |
| logistic_regression | 0.682 (0.649-0.718) | 0.251 (0.220-0.306) | 0.287 (0.272-0.300) | 0.927 (0.893-0.970) | 0.225 (0.198-0.256) | 0.324 (0.299-0.352) | 0.278 (0.246-0.309) | -2.098 (-2.291--1.975) | 0.731 (0.630-0.942) |
| random_forest | 0.686 (0.656-0.722) | 0.255 (0.219-0.305) | 0.166 (0.158-0.172) | 0.858 (0.816-0.898) | 0.373 (0.343-0.409) | 0.441 (0.415-0.471) | 0.301 (0.266-0.332) | -1.216 (-1.346--1.052) | 1.269 (1.062-1.540) |
| xgboost | 0.671 (0.642-0.712) | 0.257 (0.229-0.299) | 0.237 (0.224-0.246) | 0.938 (0.908-0.964) | 0.237 (0.205-0.276) | 0.336 (0.312-0.362) | 0.284 (0.257-0.312) | -1.836 (-1.967--1.733) | 0.702 (0.589-0.872) |

**C. Band of MAP 65–69 mmHg**

| **Model** | **AUROC**  **(95% CI)** | **AUPRC**  **(95% CI)** | **Brier Score**  **(95% CI)** | **Sensitivity**  **(95% CI)** | **Specificity**  **(95% CI)** | **Accuracy**  **(95% CI)** | **F1 Score**  **(95% CI)** | **Calibration Intercept (95% CI)** | **Calibration Slope**  **(95% CI)** |
| --- | --- | --- | --- | --- | --- | --- | --- | --- | --- |
| extra_trees | 0.660 (0.630-0.692) | 0.379 (0.333-0.430) | 0.221 (0.214-0.227) | 0.997 (0.990-1.000) | 0.016 (0.008-0.028) | 0.244 (0.222-0.265) | 0.380 (0.351-0.409) | -1.103 (-1.228--0.983) | 1.167 (0.958-1.410) |
| logistic_regression | 0.637 (0.600-0.672) | 0.363 (0.306-0.421) | 0.380 (0.365-0.397) | 0.985 (0.968-0.994) | 0.041 (0.029-0.053) | 0.260 (0.238-0.286) | 0.382 (0.354-0.411) | -1.799 (-2.006--1.569) | 0.599 (0.429-0.751) |
| random_forest | 0.661 (0.631-0.696) | 0.373 (0.323-0.431) | 0.235 (0.227-0.243) | 0.985 (0.971-0.997) | 0.050 (0.034-0.069) | 0.267 (0.244-0.286) | 0.385 (0.355-0.414) | -1.239 (-1.366--1.125) | 1.215 (0.954-1.531) |
| xgboost | 0.622 (0.591-0.657) | 0.341 (0.299-0.390) | 0.324 (0.310-0.338) | 0.963 (0.939-0.985) | 0.079 (0.062-0.101) | 0.285 (0.262-0.310) | 0.385 (0.358-0.410) | -1.486 (-1.665--1.302) | 0.497 (0.356-0.685) |

**Supplementary Table 6. External validation of model performance at the window and patient levels**

**A. Window-level performance**

| **Model** | **AUROC**  **(95% CI)** | **AUPRC**  **(95% CI)** | **Brier Score**  **(95% CI)** | **Sensitivity**  **(95% CI)** | **Specificity**  **(95% CI)** | **Accuracy**  **(95% CI)** | **F1 Score**  **(95% CI)** | **Calibration Intercept (95% CI)** | **Calibration Slope**  **(95% CI)** |
| --- | --- | --- | --- | --- | --- | --- | --- | --- | --- |
| random_forest | 0.894 (0.875-0.907) | 0.540 (0.457-0.596) | 0.092 (0.083-0.102) | 0.871 (0.835-0.898) | 0.749 (0.704-0.787) | 0.762 (0.727-0.795) | 0.441 (0.399-0.476) | -0.756 (-0.848--0.677) | 1.955 (1.820-2.079) |
| extra_trees | 0.889 (0.870-0.903) | 0.517 (0.437-0.577) | 0.089 (0.080-0.099) | 0.892 (0.860-0.918) | 0.728 (0.682-0.758) | 0.745 (0.709-0.770) | 0.430 (0.385-0.468) | -0.685 (-0.775--0.587) | 1.752 (1.632-1.896) |
| xgboost | 0.868 (0.840-0.883) | 0.472 (0.386-0.540) | 0.099 (0.088-0.112) | 0.782 (0.731-0.824) | 0.773 (0.736-0.805) | 0.774 (0.743-0.801) | 0.427 (0.372-0.470) | -1.271 (-1.395--1.186) | 0.993 (0.901-1.081) |
| logistic_regression | 0.852 (0.827-0.870) | 0.452 (0.351-0.516) | 0.161 (0.144-0.179) | 0.795 (0.731-0.835) | 0.732 (0.693-0.766) | 0.739 (0.707-0.768) | 0.396 (0.347-0.434) | -2.094 (-2.193--1.986) | 0.821 (0.761-0.895) |

**B. Patient-level performance**

| **Model** | **AUROC**  **(95% CI)** | **AUPRC**  **(95% CI)** | **Sensitivity**  **(95% CI)** | **Specificity**  **(95% CI)** | **Accuracy**  **(95% CI)** | **F1 Score**  **(95% CI)** | **Calibration Intercept**  **(95% CI)** | **Calibration Slope**  **(95% CI)** | **Mean alerts per case-hour**  **(95% CI)** |
| --- | --- | --- | --- | --- | --- | --- | --- | --- | --- |
| xgboost | 0.950 (0.916-0.977) | 0.968 (0.943-0.984) | 0.929 (0.885-0.969) | 0.772 (0.703-0.849) | 0.899 (0.871-0.931) | 0.870 (0.833-0.913) | 1.465 (1.029-2.076) | 2.499 (1.946-3.505) | 3.488 (3.028-4.004) |
| random_forest | 0.971 (0.950-0.987) | 0.982 (0.969-0.993) | 0.981 (0.958-0.997) | 0.728 (0.633-0.812) | 0.915 (0.880-0.942) | 0.886 (0.843-0.921) | 3.075 (2.351-4.435) | 5.580 (4.476-8.630) | 3.847 (3.451-4.360) |
| logistic_regression | 0.946 (0.914-0.968) | 0.966 (0.941-0.981) | 0.968 (0.943-0.993) | 0.761 (0.670-0.851) | 0.917 (0.886-0.946) | 0.890 (0.852-0.927) | -0.289 (-0.749-0.050) | 2.102 (1.727-2.644) | 3.908 (3.303-4.391) |
| extra_trees | 0.968 (0.954-0.984) | 0.980 (0.970-0.990) | 0.987 (0.967-1.000) | 0.750 (0.665-0.842) | 0.924 (0.899-0.952) | 0.898 (0.866-0.935) | 3.478 (2.757-5.260) | 4.641 (3.895-6.649) | 4.094 (3.599-4.668) |
